# "They are our eyes outside there in the community": Implementing enhanced training, management and monitoring of South Africa’s ward-based primary healthcare outreach teams

**DOI:** 10.1371/journal.pone.0266445

**Published:** 2022-08-26

**Authors:** Joanne E. Mantell, Tsitsi B. Masvawure, Jennifer M. Zech, William Reidy, Martin Msukwa, Mary Glenshaw, Jonathan Grund, Daniel Williams, Blanche Pitt, Miriam Rabkin

**Affiliations:** 1 HIV Center for Clinical and Behavioral Studies, Gender, Sexuality and Health Area, New York State Psychiatric Institute and Department of Psychiatry, Columbia University Irving Medical Center, New York, New York, United States of America; 2 Health Studies Program, Center for Interdisciplinary Studies, College of the Holy Cross, Worcester, Massachusetts, United States of America; 3 ICAP at Columbia University, New York, New York, United States of America; 4 ICAP South Africa, Pretoria, South Africa; 5 Division of Global HIV & TB, Centers for Disease Control and Prevention, Center for Global Health, Pretoria, South Africa; 6 Departments of Medicine and Epidemiology, Columbia University Mailman School of Public Health, New York, New York, United States of America; University of Cape Town Faculty of Science, SOUTH AFRICA

## Abstract

**Introduction:**

In 2018, South Africa’s National Department of Health provided additional resources for ward-based primary healthcare outreach teams (OT) with support from the U.S. President’s Emergency Plan for AIDS Relief. The intervention package included a new training curriculum, enhanced staffing, revised management and supervisory structures, and more intensive monitoring and evaluation (M&E). The goal was to strengthen OT and their impact on both primary healthcare and HIV-specific services. We conducted a process evaluation of this intervention package during its second year and examined implementation successes and challenges.

**Methods:**

We conducted a mixed-methods evaluation at 20 purposively selected facilities in Bojanala and City of Tshwane districts, including surveys with 222 community health workers (CHWs) and outreach team leaders (OTLs); key informant interviews and online surveys with 28 policy and program stakeholders; 70 in-depth interviews with health facility staff; 20 focus group discussions with 194 CHWs; 20 structured health facility assessments; directly-observed time-motion studies; and review of program documents.

**Results:**

Most participants highlighted the hiring and training of CHWs and OTLs as a key implementation success because this had partially alleviated staffing shortages and helped clarify CHWs’ and OTLs’ responsibilities and supervisory structures. The new monitoring tools were welcomed for their potential to improve data collection and program tracking. However, participants highlighted many program challenges: short-lived gains in CHWs’ knowledge and skills due to lack of ongoing training and mentoring; insufficient integration of OT into health facility management structures; persistent shortages of equipment, supplies, transportation, and workspace for CHWs; and insufficient remuneration for staff.

**Conclusion:**

Strengthening and expanding CHW programs, such as OT, requires intensive support and continuous investments. To sustain improvements in training, supervision, and job satisfaction, CHWs must be equipped with needed resources, provided with ongoing supportive supervision, and strengthened by optimized program management, monitoring and processes.

## Introduction

Community health workers (CHWs) are widely used in resource-constrained and middle-income countries to provide primary healthcare (PHC) services and bridge the gap between health facilities and communities in the context of healthcare worker shortages. They are also seen as a key contributor to the attainment of health-related Sustainable Development Goals 3 and 6 [1] by acting as community mobilizers, health promoters and providing preventive and clinical services [2, 3]. South Africa’s rich history of CHW programs is rooted in the 1930s with the training of malaria assistants and the establishment of the Pholela Health Center in 1940 [4], which led to the training of CHWs in infectious diseases control and community health education [5]. CHW roles have included a wide range of grassroots activities, including health promotion, health education, community health assessments, and linkage to services for maternal and child health, tuberculosis, HIV, and other chronic conditions. Over time, a growing number of small-scale CHW programs were implemented by non-governmental and faith-based organizations [6].

The district health system adopted by South Africa’s first democratic government in 1994 did not include CHWs, but a framework was created in 2004 to support the development of a national CHW program [7]. This was formalized in 2010, when ward-based PHC outreach teams (OTs) were created as part of South Africa’s “Re-engineering of Primary Health Care” strategy [8], building on the long history of initiatives to culminate in the national CHW program designed to link health facilities and community-based health services [4]. Between 2010 and 2017, CHWs had a supervisory structure that included health facility-based professional nurses designated as Outreach Team Leaders (OTLs). CHWs and their OTLs were organized into OT to provide integrated PHC services to individuals, families and households within specified geographic areas, officially known as Ward-Based Primary Healthcare Outreach Teams [8]. However, due to high workloads and a paucity of professional nurses to serve as OTLs, implementation of this structure varied widely throughout the country. In 2018, in practice, many CHWs still reported directly to facility managers in the majority of facilities, and few were formally supervised [8].

In 2018, South Africa’s National Department of Health (NDoH) and the U.S. President’s Emergency Plan for AIDS Relief (PEPFAR) announced a USD1.2 billion increase in funding and activities aimed at identifying an additional two million people living with HIV in South Africa and initiating them on antiretroviral therapy (ART) [9]. In support of this surge, PEPFAR committed over USD50 million to expand CHW services and strengthen OT programs to enhance their impact on HIV testing, linkage to treatment, and retention in care.

The inclusion of OT in the provision of HIV service delivery was a purposeful attempt to improve access to those services and bridge the gap between facility-based and community-based HIV programs [8]. Since CHWs are usually members of the communities they serve, they have an opportunity to develop sustained relationships with recipients of care and are often trusted sources of information about HIV testing, prevention, and treatment [4, 10–12] as well as generally influential in the lives of community members [13]. This is key to the effective promotion of ART adherence, linkage of people to nearby health facilities for HIV-related services, and follow-up for missed appointments [14, 15].

Prior to 2018, known challenges related to OT effectiveness included inconsistent supervision, sub-optimal integration into health facility systems, overly broad CHW scopes of work and limited CHW in-depth knowledge and skills [14, 16, 17]. In response, the NDoH and PEPFAR collaborated to design and implement a “surge” of intensified support for the OT program with four key components: 1) staffing, 2) training, 3) management, and 4) monitoring and evaluation (M&E).

NDoH and PEPFAR support for staffing included defining optimal CHW/OTL ratios per team and formalizing the shift from professional nurses to enrolled nurses as OTLs as outlined in the *NDoH Policy Framework and Strategy for Ward Based Primary Healthcare Outreach Teams 2018/19–2023/24* [8]. This change was intended to foster more cost-effective OT management, enable role distinction among nurses, and create positions with expanded work roles and service delivery focused on households. Staffing support included determining the number of teams needed based on the HIV burden and catchment size of health facilities. The Policy Framework supports a scaled-up approach to allow for increased numbers of CHWs as additional funding becomes available, prioritizing the poorest communities and aiming for geographic equity [8].

The training intervention, supported by the NDoH and PEPFAR (through the U.S. Centers for Disease Control and Prevention (CDC) South Africa) and conducted by the International Training and Education Center for Health (I-TECH), included new competency-based curricula for OTLs and CHWs. The new training program focused on the most prevalent health conditions in the community, including HIV/AIDS. OTL training comprised a written pre-test knowledge assessment and seven days of didactic classroom education to enhance cross-cutting skills in ethics and confidentiality, communication, health promotion, screening, referrals, tracing, psychosocial support, M&E, supportive supervision, and mentoring. This was followed by eight weeks of field-based training and a second skills assessment. CHW training had a similar structure: pre-test knowledge assessment, five days of classroom skills training on key clinical topics, followed by nine weeks of on-site practical training and a plan for ongoing supportive supervision and refresher trainings. CHW core skills were assessed using a post-test knowledge assessment (repeat of pre-test). In, A total of 1,664 OTLs and 22,216 CHWs were trained using the new curriculum as of September 2019. Facility managers also received a one-day orientation which outlined the role of CHWs, OTLs and facility managers and the contribution of OT activities to reaching PHC targets.

In addition to the enhanced staffing and training, new M&E tools and systems were developed to track OT activities at the team and individual levels. The M&E tools were developed by NDoH in collaboration with I-TECH and included CHW daily and weekly summary activity sheets, household record forms, and OTL monthly activity summary forms. Paper-based M&E tools were piloted in two districts and adapted to an electronic mHealth platform to improve the efficiency and quality of data collection, inform patient and program management, and importantly, improve linkage to care and early identification of loss to follow-up.

In 2019, ICAP at Columbia University conducted a formative process evaluation of the OT surge in partnership with the NDoH and PEPFAR. The evaluation was designed to be immediately policy-relevant and to provide rapid mid-program feedback. This paper examines program implementation and barriers and successes from the perspectives of the NDoH, implementing partners, facility-level staff, and the OT.

## Methods

### Study design

**Fig 1:**
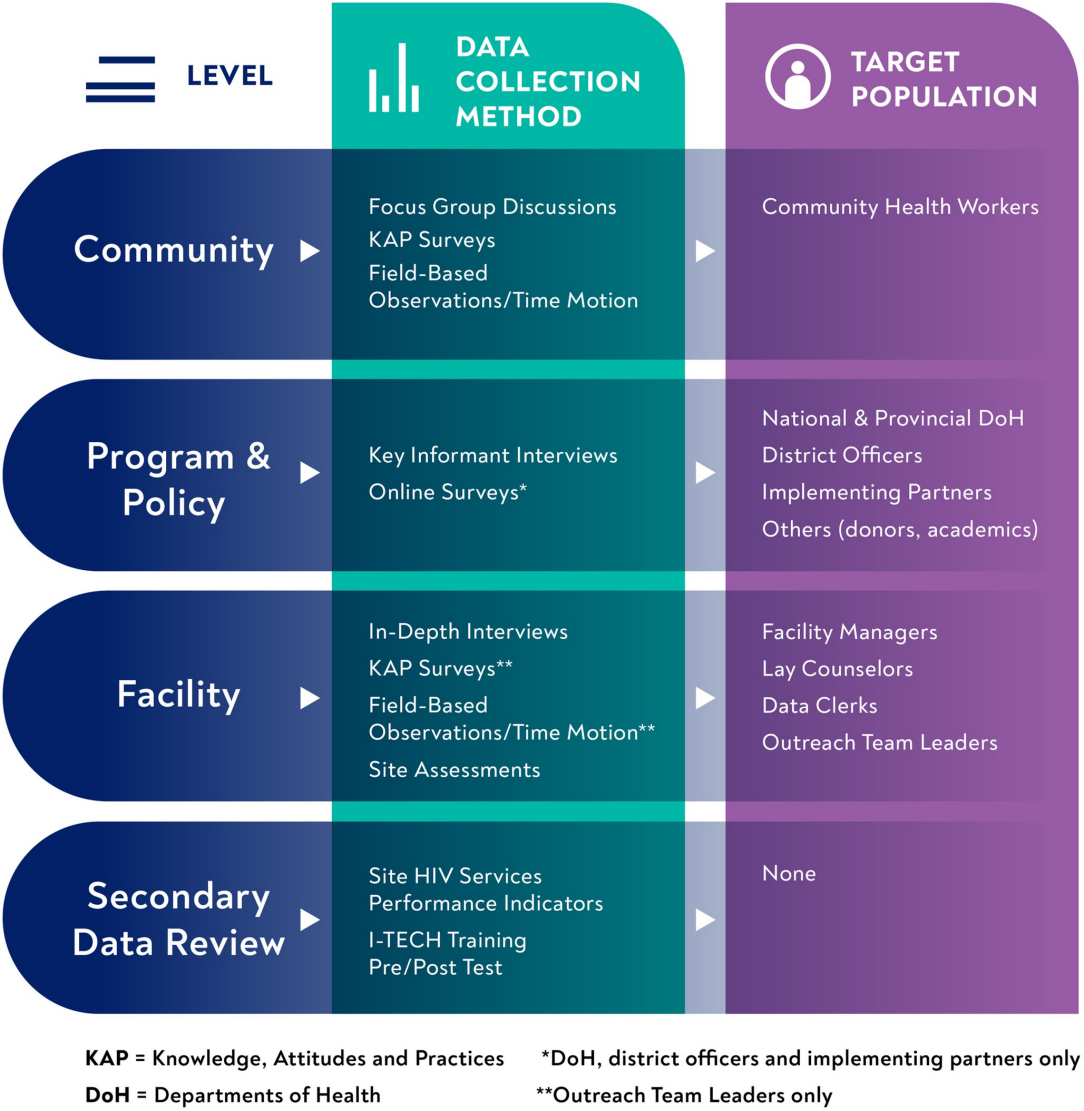
Multi-level, multi-method concurrent data collection. KAP, Knowledge, Attitudes and Practices; DoH, Department of Health *DoH, district officers and implementing partners only ** Outreach Team Leaders only.

The process evaluation, conducted between September and November 2019, used a parallel convergent mixed-methods design, with concurrent collection of qualitative and quantitative data at multiple levels (see Fig 1). Health facility and community-based data were collected at 20 public-sector PHC facilities and their catchment areas in two districts receiving intensive support from PEPFAR—the City of Tshwane and Bojanala in Gauteng and North West provinces, respectively. We also reviewed relevant program documents, e.g., the Policy Framework and Strategy for Ward Based Primary Healthcare Outreach Teams 2018/19–2023/24 [8], OTL and CHW training manuals, and South Africa’s National Strategic Plan on HIV, TB and STIs 2017–2022 [18]. We triangulated both methods and data sources, a research strategy to test validity through the convergence of information, overcome limitations of using any one method or source, and gain new insights about program implementation [19–21].

### Sampling: Health facilities and participants

Districts were purposively selected in partnership with the NDoH and PEPFAR based on the size of their HIV programs, higher HIV prevalence, and representation of urban, peri-urban, and rural facilities. Ten health facilities in each district were purposively selected in partnership with district management teams and implementing partners based on the following criteria: 1) staffing levels (≥3 CHWs and one OTL employed and currently assigned to an OT); 2) number of individuals receiving ART at those health facilities (900–4000, middle two quartiles of district health facilities); 3) HIV program performance in the middle two quartiles of district health facilities using selected PEPFAR Monitoring, Evaluation and Reporting (MER) indicators for HIV linkage, treatment, retention based on October 2018-September 2019 data reported to PEPFAR; and 4) type of setting (urban, peri-urban, and rural, as defined by NDoH). We excluded hospitals, private sector health facilities, and health facilities implementing research projects or other/non-standard interventions related to OT.

We used purposive sampling, followed by snowball sampling, to recruit key informant interview (KII), in-depth interview (IDI), and online survey participants. We selected policy and program stakeholders with experience in the expanded program and health facility staff who worked directly with OT, such as facility managers, or those who were otherwise familiar with the program, such as data clerks and lay counselors. Facility managers assisted with the recruitment of CHWs and OTLs for the knowledge, attitude and practice (KAP) surveys and focus group discussions (FGDs).

### Data collection methods

#### Qualitative methods

*KIIs with policy and program stakeholders and IDIs with facility-level staff*. Semi-structured interview guides for both KIIs and IDIs focused on participants’ perceptions of the key successes and challenges of the expanded program. Each interview took approximately one hour, was digitally recorded, and was conducted in English (all participants speak English) by a trained interviewer in a private space at the participant’s work site or via telephone.

*FGDs with CHWs on OT*. These explored CHWs’ views of, and experiences with, various components of the expanded program, such as training, supervision, management, and household visits. Each FGD took approximately two hours, was digitally recorded, had 6–10 participants, was conducted in one of three local languages (*i*.*e*., Setswana, Sepedi and IsiZulu) by two trained interviewers, and was held in a private location on the health facility premises with no supervisors present.

#### Quantitative methods

*Online survey with policy and program stakeholders*. Policy and program stakeholders who were unavailable for an in-person interview completed a self-administered 15-minute online survey, which assessed perceptions of the key successes, innovations and challenges of the expanded program. The 22-item survey was designed in Qualtrics (Version September 2019, Qualtrics International Inc., Provo, UT, USA), an online survey tool.

*KAP surveys with CHWs and OTLs*. The survey was conducted in September and October 2019 with a subset of the 588 OTLs and CHWs who had received training on the new curriculum at the 20 participating facilities. It included questions about satisfaction with training and supervision and was the same test administered by I-TECH before and after the 2018–2019 training to assess longer-term knowledge retention. Survey questions were essentially the same for OTLs and CHWs; however, OTLs were asked additional questions about their supervisory roles, and CHWs were asked additional questions about household data collection. Surveys were self-administered, took an average of 45 minutes, and were conducted in English.

*OT field-based observations/time-motion study*. We conducted field-based observations/time-motion studies of OT from the 20 participating health facilities as they conducted their daily home visits, meetings, and activities/campaigns. Trained study staff, usually working in pairs, accompanied each team and collected time-motion data (*e*.*g*., travel time from a health facility to a patient’s home and duration of a home visit) and documented key activities (*e*.*g*., routine daily home visits and meetings as well as challenges and successes) using a structured observation checklist. At the health facilities, study staff observed planning meetings between OTLs and CHWs and documented other facility-based activities. Each observation took two hours on average.

*Site assessments*. These were conducted by trained study staff at each participating health facility. The structured site assessments to document availability and data quality included: 1) review of key HIV and expanded program data, such as staffing levels, training, and management and M&E systems, 2) discussion with facility managers about the expanded program, and 3) review of OT data collection forms, including CHW Activity Sheets, CHW Weekly & Summary Sheets, OTL Monthly Summary Forms, and Supervisor Assessment Forms.

#### Secondary data review

*Secondary review of HIV services data*. We reviewed the MER indicators from U.S. Fiscal Year 2019 from the PEPFAR database (Data for Accountability, Transparency, and Impact Monitoring [DATIM]) to characterize HIV linkage, treatment, and retention for the 20 participating sites.

*Secondary review of previous pre-and post-test training data*. To assess knowledge retention among the trained OTLs and CHWs, we compared aggregate results of the three repeated surveys. The first and second were pre- and post-test training results from I-TECH-administered surveys of all CHWs and OTLs at the 20 participating sites between August 2018 and July 2019, and the third, which used identical questions, was included in the KAP surveys (see below) that we administered to all trained CHWs and OTLs working on OT at the participating sites during data collection (September-November 2019), a subset of the CHWs and OTLs who were originally trained.

S1 Table in the supplementary document presents the data collection strategy by sample and assessment domains.

### Data management and analysis

KIIs, IDIs, and FGDs were digitally recorded and transcribed. IDIs and FGDs were translated by bilingual senior research staff fluent in Setswana, Sepedi, and/or isiZulu, as well as English. All transcripts were reviewed for accuracy and completeness prior to analysis. Qualitative data were entered and coded by question using the Dedoose^™^ Software Package (Version 8.1.8, SocioCultural Research Consultants, LLC, Los Angeles, CA, USA). Two researchers coded each transcript and met frequently to compare and reconcile the application of codes. Data were analyzed using thematic content analysis, summarized, and shared with other study team members for review. A team of five researchers worked together to reach consensus on codes and data interpretation and used framework analysis to organize the data by the successes and challenges of program implementation of each expanded OT activity [22].

Quantitative data from the health facility assessments, KAP surveys and field observations were collected on tablets and uploaded to a central SurveyCTO^™^ server (Version 2.60, Dobility, Inc. Cambridge, MA, USA), with internal data quality checks conducted to identify valid entries, skip patterns, and missing values. The online survey data were collected via the Qualtrics platform. Data were downloaded from the SurveyCTO^™^ and Qualtrics, cleaned, and analyzed using SAS (Version 9.4, SAS Institute Inc., Cary, NC, USA). Descriptive analyses were conducted for data from the health facility assessments, KAP surveys, field observations, and online surveys. We reported numbers, percentages, means, medians and ranges whenever appropriate.

The KAP survey score was measured as the proportion of correct answers for each participant. Questions with multiple correct responses were considered to be answered correctly only when *all* correct response options were selected. The mean proportions of correct answers between pre-test and post-test were compared using a paired t test, which was used for comparison between post-test scores and repeat test scores because unique identification numbers were not recorded in the same way in the two databases. The analyses were stratified by district and cadre (CHW vs. OTL).

All data were de-identified and sent electronically to the research team for analysis in accordance with the Health Protection Portability and Accountability Act (HIPAA).

### Ethical review

The evaluation protocol was approved by the Institutional Review Boards/Ethics Committees at the Columbia University Irving Medical Center (protocol IRB-AAAR9955), Human Sciences Research Council (HSRC) in South Africa (protocol-REC 2/18/07/18), and the CDC (protocol 2019–171). The project was reviewed in accordance with CDC human research protection procedures and was determined to be research, but CDC investigators did not interact with human subjects or have access to identifiable data or specimens for research purposes. The South Africa NDoH, provincial and district health offices, and the U.S Health Resources and Services Administration (HRSA) provided written approval. The study was submitted to the National Department of Health Research Database repository (GP_201907_027 and NW_201907_001). All participants provided written informed consent prior to study participation. No compensation was provided to study participants.

## Results

### Site characteristics

Ten sites were located in Bojanala, a district in North West province about 182km from Johannesburg; and 10 sites were located in City of Tshwane, a district in Gauteng province about 50km from Johannesburg. All 20 participating sites were PHC facilities; their characteristics are described in Table 1 based on data from site assessments. Table 2, a snapshot of the PEPFAR MER indicators, provides context on HIV services at the time of the study; it is not an assessment of program impact. Half of the sites were rural and the other half either urban (n = 7) or peri-urban (n = 3). A median of two OTLs were active per site (range = 1–4) and 74% of active OTLs had been trained on the new curriculum. Sites had a median of 25 active CHWs (mean = 26, range = 3–58), 77% of whom had been trained on the new curriculum. Fourteen health facilities had written standard operating procedures for M&E, 10 had M&E-related job aids, and 12 relied exclusively on paper-based, rather than electronic, M&E tools for documenting and tracking community-based services.

**Table 1:** Site characteristics of the 20 evaluation facilities, September-November 2019.

		Bojanala, n = 10 n (%)	Tshwane, n = 10 n (%)	All sites, n = 20 n (%)
Setting	Urban	1 (10)	6 (60)	7 (35)
	Peri-urban	1 (10)	2 (20)	3 (15)
	Rural	8 (80)	2 (20)	10 (50)
Number of patients on ART	Median	1635	2724.5	2152
	Range	1072–5743	657–4871	657–5743
	IQR	1220, 2175	2128, 4155	1272, 3103
Number of patients with VL in last 6 months	Median	599	751.5	599
	Range	18–1023	23–2459	18–2459
	IQR	449, 772	350, 1382	428, 1039
Number of patients with VL <400 copies in past 6 months	Median	533	771	595.5
	Range	13–906	23–2199	13–2199
	IQR	397, 631	210, 1242	303.5, 909.5
Outreach Team training date range	Range (month/year)	Sept 2018- May 2019	Aug 2018- July 2019	Aug 2018- July 2019
Number of OTLs actively working on Outreach Teams	Median	2	3	2
	Range	1–3	1–4	1–4
	IQR	1, 3	2, 4	2, 3
Number of CHWs actively working on Outreach Teams	Median	16	35	24.5
	Range	7–58	3–52	3–58
	IQR	14, 26	24, 41	14.5, 36

ART = antiretroviral therapy

VL = viral load

OTLs = Outreach Team Leaders

CHWs = Community Health Workers

**Table 2:** HIV performance indicators for the 20 evaluation facilities, median (IQR) of annual mean of quarterly results per facility, October 2018-September 2019*.

	Bojanala, n = 10	Tshwane, n = 10	All sites, n = 20
Median Indicator	Median (IQR)	Median (IQR)	Median (IQR)
Patients testing HIV positive, per quarter	60 (42,75)	140 (111,180)	85 (64,140)
Patients initiating ART, per quarter	55 (36,65)	153 (107,191)	84 (58,153)
Patients currently on ART**	1254 (1189,1886)	2814 (2289, 3780)	1958 (1225, 2757)
Percent VL testing coverage proxy***	73% (70%,78%)	73% (68%,79%)	73% (69%,79%)
Percent of patients with VL suppression among those tested	92% (92%,93%)	94% (92%,95%)	93% (92%,95%)

ART = antiretroviral therapy

VL = viral load

*Data source: PEPFAR Monitoring, Evaluation and Reporting (MER) data, fiscal year 2019

**At year midpoint (end of Q2)

***Median of mean quarterly proxy VL coverage per facility, which was calculated as the number of current ART patients receiving a VL test in the past 12 months divided by the number of current ART patients

### Sample characteristics

**Fig 2:**
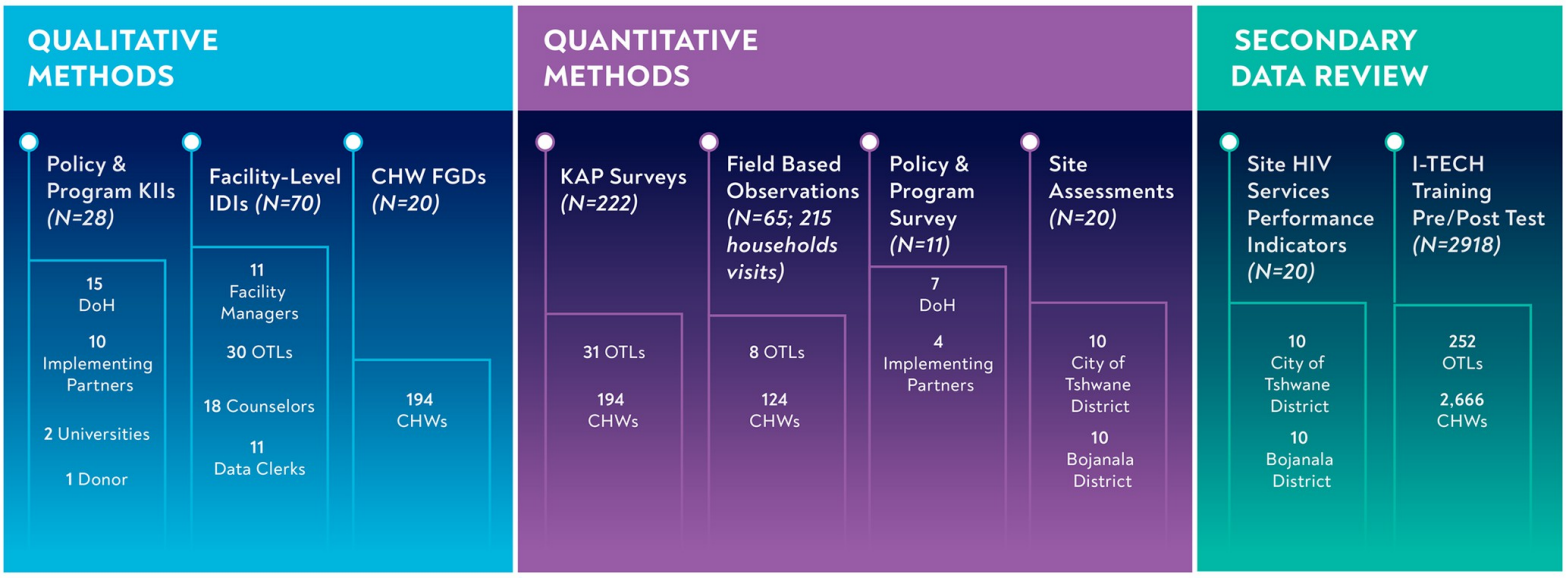
Sample size for each data collection method. KIIs = Key Informant Interviews DoH = Department of Health IDIs = In-depth Interviews CHWs = Community Health Workers FGDs = Focus Group Discussions KAP = Knowledge, Attitudes and Practices.

We engaged 657 participants in the study. Below, we present the sample size (Fig 2) and demographic characteristics of the sample for each for each data source (Table 3).

**Table 3:** Participant characteristics by data collection method*.

		Policy and Program Stakeholders n = 39		Facility-level IDIs n = 70 n (%)	CHW FGDs n = 194 n (%)	KAP Survey n = 222	
		KIIs n = 28 n (%)	Online Surveyn = 11 n (%)			CHW n = 191 n (%)	OTL n = 31 n (%)
Sex	Male	4 (14)	2 (18)	11 (16)	9 (5)	6 (3)	4 (13)
	Female	24 (86)	9 (82)	58 (83)	183 (94)	185 (97)	26 (84)
	Decline to answer	0 (0)	0 (0)	1 (1)	2 (1)	0 (0)	1 (3)
Age	Median	51	47.5	39	40	40	44
	Range	32–75	32–57	27–67	22–65	22–60	27–68
	IQR	40, 54	35, 55	32, 54	32, 46	32, 46	33, 64
Highest education level	Some primary	0 (0)	0 (0)	0 (0)	-	2 (1)	0 (0)
	Completed primary	0 (0)	0 (0)	0 (0)	-	4 (2)	0 (0)
	Some secondary	0 (0)	0 (0)	1 (1)	-	51 (27)	1 (3)
	Completed secondary	0 (0)	2 (18)	18 (26)	-	112 (59)	7 (23)
	Some tertiary	1 (4)	0 (0)	15 (21)	-	15 (8)	7 (23)
	Completed tertiary	27 (96)	9 (82)	36 (51)	-	3 (2)	14 (45)
	Decline to answer	0 (0)	0 (0)	0 (0)	-	4 (2)	2 (6)
Work location	National	3 (11)	1 (10)	-	-	-	-
	Provincial	25 (89)	3 (27)	4 (6)	-	-	-
	District	-	7 (64)	66 (94)	194 (100)	191 (100)	31 (100)
	Bojanala	-	3 (27)	39 (56)	85 (44)	93 (49)	14 (45)
	City of Tshwane	-	4 (36)	27 (39)	109 (56)	98 (51)	17 (55)
Organization/ role	DoH	15 (54)	7 (64)	-	-	-	-
	Implementing partner	10 (36)	4 (36)	-	-	-	-
	Funding & training institution	3 (11)	0 (0)	-	-	-	-
	Facility manager	-	-	11 (16)	-	-	-
	OTL	-	-	30 (43)	-	-	31 (100)
	Counselor	-	-	18 (26)	-	-	-
	Data clerk	-	-	11 (16)	-	-	-
	CHW	-	-	-	194 (100)	191 (100)	-
Years working with/on Outreach Teams	< 1 year	5 (18)	1 (10)	5 (7)	22 (11)	36 (19)	7 (23)
	1–5 years	10 (36)	5 (45)	41 (59)	101 (52)	80 (42)	23 (75)
	6–10 years	9 (32)	5 (45)	16 (23)	59 (30)	74 (39)	1 (3)
	> 10 years	3 (11)	0 (0)	7 (10)	12 (6)	1 (1)	0 (0)
	Declined to answer	1 (4)	0 (0)	1 (1)	0 (0)	0 (0)	0 (0)
Years working as CHW or OTL	< 1 year	-	-	-	0 (0)	2 (1)	0 (0)
	1–5 years	-	-	-	40 (21)	33 (17)	9 (29)
	6–10 years	-	-	-	75 (39)	84 (44)	9 (29)
	> 10 years	-	-	-	77 (40)	70 (37)	12 (39)
	Declined to answer	-	-	-	2 (1)	2 (1)	1 (3)

KIIs = Key Informant Interviews

DoH = Department of Health

IDIs = In-depth Interviews

CHWs = Community Health Workers

FGDs = Focus Group Discussions

KAP = Knowledge, Attitudes and Practices

* Dash (-) = NA

### Emergent themes: Perceptions and experiences of core elements of the expanded OT program

**Fig 3:**
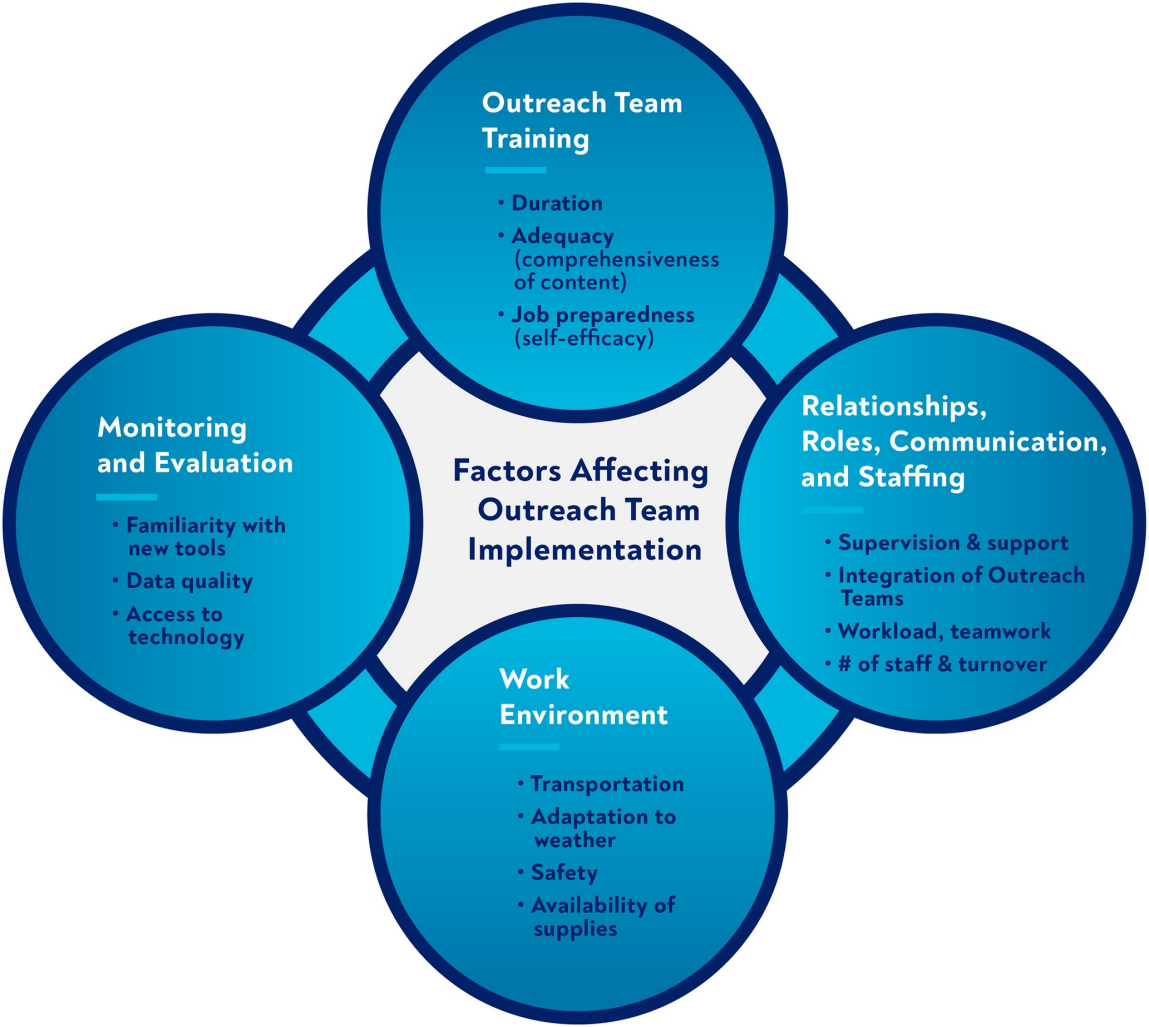
Key factors affecting implementation of expanded outreach team activities.

There was more consensus on the strengths and challenges of the program than there were differences among participant cadres. Four main interrelated themes emerged: 1) appreciation for the newly-designed training and recognition of the importance of ongoing supportive supervision; 2) concerns related to the relationship between teams and health facility management structures, roles, communication and staffing; 3) importance of enhancing the work environment for CHWs and OTLs at both community and facility levels; and 4) early challenges with implementation of the new M&E strategy. Within each theme, we identified key factors affecting implementation of expanded activities, and describe participants’ perceptions of the strengths and challenges of the expanded program (Fig 3).

#### OT training

As described above, the new training curriculum for CHWs and OTLs was the foundation of the intensified expanded program. The competency-based training was aligned with the revised scope of work for each cadre and focused on building new skills to improve CHWs’ task performance, relations with the community, communication and to enhance OTLs’ supervisory skills.

*Perceived strengths*. In the quantitative surveys, 77% (n = 147/191) of CHWs and 94% (n = 29/31) of OTLs indicated that the training had either prepared them “completely” or “very well” for their current roles. Additionally, 91% (n = 10/11) of policy and program stakeholders highlighted the training as an important success of the expanded program. Qualitative interview and FGD data also suggest that the training increased CHWs’ and OTLs’ confidence in their ability to carry out tasks professionally and improved understanding of their roles, including supervisory and reporting expectations.

*…now we know how to do our jobs*. *Before we were in the dark*. *We were just going out with the CHW without knowing what we were going to do*, *just hi-jacking the things*, *but now we know when we said we’re going to do the tracing*, *we know what we are going to do*. *And then what is the supervised visit*, *what are we going to do*. *We know now*.

–OTL, IDI

Similarly, the benefits of training were supported by the KIIs, with facility managers and policy and program stakeholders attributing improvements in knowledge and role clarification to the CHWs and OTLs being educated on the new standardized scope of work and equipped with essential skills to carry out a diverse range of activities.

*I think the last training that we had was focused on soft skills…communication*, *health education*, *referral*, *screening*, *tracing and psycho-social…to equip the team leaders on how to manage*, *how to supervise*, *how to do M&E…I think that is the most important success that we achieve now*.

–Policy and program stakeholder, KII

All participant cadres also appreciated the large number of CHWs and OTLs hired and trained, as this was seen as easing staff shortages.

*…in some clinics you find that there is one OTL versus 30 CHWs but now they are bringing more so maybe it will be 1*:*10*, *which is 1 OTL versus 10 CHWs*. *At least now they are trying their best*… *the district and then of course my organization has got those OTLs*, *l think we have got 14 if not 15 in the entire district which is* [anonymous].

–Policy and program stakeholder, KII

*Perceived challenges*. Most CHWs felt the training was too short considering the extensive amount of information covered. They highlighted continuing gaps in their knowledge (e.g., regarding medications and new policies) and recommended extending the length of the training to cover topics in greater detail.

*Sometimes they might give us medication that we don’t know and when we get to our patient*, *she will ask me what it is*, *and I won’t even know how to explain it…*. *Sometimes it ruins our reputation…when I get to the office*, *my team leader doesn’t know too*. *We need more* [training] *as we work with medicine…*

–CHW, FGD

KII, IDI and FGD participants also reported that some CHWs did not receive training in all aspects of the new curriculum. Additionally, although the training implementation plan included ongoing supportive supervision and refresher trainings, these activities had not been implemented at all sites at the time of the evaluation. CHWs suggested continual in-service training and supportive supervision to advance their skills and stay abreast on new health information, procedures, and medications.

**Fig 4:**
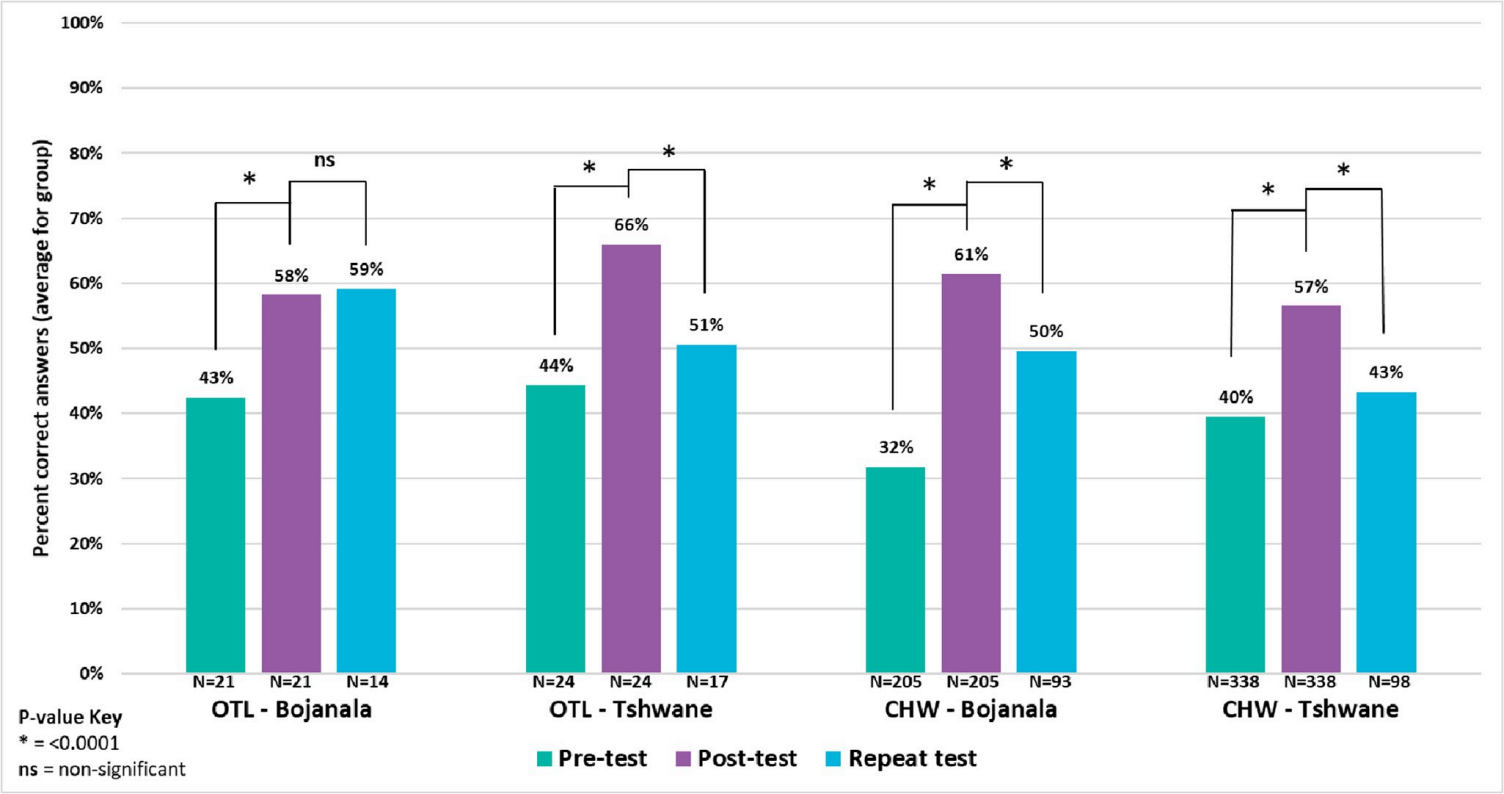
CHW and OTL test of knowledge: Before training, immediately post-training and 3–12 months post-training by health district. OTL = Outreach Team Leader CHW = Community Health Worker.

The trainees’ perception that their training was too brief to retain knowledge was echoed by the results of their repeat testing in the KAP survey. Fig 4 compares the pre- and post-test training results from the 588 CHWs and OTLs who received training in 2018–2019 at the 20 participating sites with the results of the repeat test administered to a subset of these trainees (i.e., the 222 trained CHWs and OTLs who participated in the KAP survey) in 2019. In both districts, OTLs and CHWs test scores improved between the pre-test and post-test (p<0.0001) at the time of training. Months later, repeat OTL testing scores showed sustained performance in Bojanala (p = 0.0533) but a drop of 15% in Tshwane (p<0.0001). CHW performance was significantly lower in both districts (-11% and -14%, respectively, p<0.0001). Both the qualitative and quantitative data were in concordance regarding training challenges faced by the expanded program.

#### Relationships, supervision, and staffing

A second major theme was the managerial relationship between the OT and health facility staff, including challenges with role clarity, communication, and staffing levels.

*Perceived strengths*. All participant cadres highlighted the recent introduction of a clear management structure for the teams as another key strength of the program. The quantitative data showed that most CHWs and OTLs had reported having a written job description detailing their roles and responsibilities (83%; n = 158/191 and 94%; n = 29/31, respectively), received a formal evaluation of their OT work in the past 12 months (73%; n = 139/191 and 81%; n = 25/31, respectively) and “strongly” or “somewhat” agreed when asked if they “consistently had the supervision needed to perform their duties” (82%; n = 157/191 and 68%;n = 21/31, respectively). Furthermore, most OTLs (84%; n = 26/31) had conducted formal evaluations of CHWs.

These survey findings were reinforced by the qualitative data. In IDIs, OTLs reported that facility managers checked in with them to ensure they had the needed resources to lead their teams and that it was easy to reach their managers and have concerns addressed in a timely manner.

*Sometimes even if she doesn’t come to us*, *she phones us even if we are the one having a problem*, *any time we can access a phone*, *call her*, *or make SMS*, *she’ll respond… and she comes to us and visits us any time*.

–OTL, IDI

Additionally, both OTLs and CHWs described their relationships and communication with each other as being good overall, despite some previous tension over supervisory styles. CHWs noted that relationships had improved after the expansion, especially after OTLs received supervisory training.

*I think our relationship with them has improved because there were times when they would speak to us in a hurtful way*, *but afterwards* [i.e., after training] *we would go to them and have a conversation that the way you spoke to us was hurtful and we did not like it*. *Then we all apologize*, *and we move on*.

–CHW, FGD

OTLs, in turn, reported that any problems that emerged were effectively addressed at team meetings and that the teams worked well together.

*The relationship is like my relationship with my kids in my household*. *Sometimes we are happy*, *sometimes we fight*, *sometimes we eat together*, *sometimes we don’t… they also say what doesn’t make them happy*. *So work is like my household*. *The nice thing is*, *we always talk to each other*. *There’s never been a time when we are so mad about each other that we don’t talk*. *It’s like our household*, *we strive for peace every day*.

–OTL, IDI

Finally, CHWs also prided themselves on teamwork, explaining that they supported each other:

*‘We have team spirit*. *When one needs help in the group*, *we help out and not belittle the person*. *We don’t point fingers asking why the person doesn’t know*. *We work in unity*. *We discuss*, *come up with a solution*, *and work carries on*.

–CHW, FGD

*Perceived challenges*. Despite the generally positive experiences around supervision, communication challenges between CHWs and OTLs emerged as an ongoing concern among CHWs. CHWs characterized some OTLs as having poor communication skills and lacking tact and reported being talked down to and shouted at.

*Some* [OTLs] *are not okay*, *honestly*. *Sometimes we put so much effort in our work … and then be told you didn’t do a great job*. *That’s very hurtful*. *It affects us to a point of feeling like quitting our work*.

–CHW, FGD

Integration of OTLs into the overall health system was reported across all participant cadres as a key challenge. Policy and program-level stakeholders in both the KIIs and survey noted that the teams were poorly integrated into the health facilities at the beginning of the expanded program, and that this had contributed to a lack of adequate support, such as dedicated resources and workspace, from health facility leadership.

*The facility managers they just don’t understand it* [Outreach Teams], *and they don’t want to hear about it*… [they] *will always prioritize what’s happening inside the clinic*, *they will tell you that ‘l’m a clinical person’ and all those things…if we can* [only] *get full buy-in from the clinic managers…*.

–Policy and program stakeholder, KII

*‘*Many CHWs and OTLs, in turn, considered the lack of dedicated workspace and resources as an indication of their overall marginalization and under-appreciation as healthcare workers. They suggested that having a workspace in the clinics would heighten recognition of the important role that the teams play in service delivery and also help improve their relationships with facility staff.

*The problem is that we don’t have a place where we say this is our home*, *our health post*. *We are squatting wherever we are…and then we walk miles*.

—OTL, IDI

Policy and program stakeholders attributed lack of integration to the generally fragmented management of PHC services in the country, whereas CHWs attributed it to their work in the community not being immediately visible to health facility staff.

*… the outreach teams are managed by* [a] *different manager while the facility that the team report to is managed by another*. *This leaves them without resources as they belong to a different cost center…*.

–Policy and program stakeholder, Online Survey

*…we are not recognized for the work we do…they say the community health workers from* [clinic] *are not working*, *whereas the patients themselves commend what we do but some members of the staff at the clinic do not commend us*. *There is no recognition*.

–CHW, FGD

Ongoing staffing challenges emerged in both the qualitative and quantitative data as additional examples of the poor integration of OTLs into health facilities. In the KAP survey, only 58% (n = 18/31) of OTLs and 66% (n = 127/191) of CHWs “strongly” or “somewhat” agreed that their workload was manageable. In the IDIs, OTLs reported that team sizes were too large for them to manage and supervise adequately, with some reporting having as many as 50 CHWs on their teams while the recommended number is 6–10 CHWs per OTL.

*Sometimes l feel like there is too much community care workers*, *sometimes the load*… *you feel that you are supervising many people… lot of people is difficult to manage compared to small number*.

–OTL, IDI

CHWs further noted that OTLs were unevenly distributed across health facilities, which meant that they did not always receive the supervision they needed.

*…it’s not enough because at times you go to a certain patient*, *and you find the situation too difficult*, *and you end up wishing that you could have gone with the supervisor*. *Like with a patient that suffers from TB MDR*, *you would wish that your supervisor could come with you so that he could advise you because we don’t have more knowledge*. *And without the supervisor*, *we might get infected*.

–CHW, FGD

Understaffing at some health facilities led to demanding workloads, especially when OTLs were asked to support non-OT-related clinic work. During the field-based observations, research staff noted that only 12% (n = 8/65) of team visits had an OTL present. The field observations also showed that limited time was allotted to logistics planning and preparing for the day with the team and OTLs. Logistics planning occurred in only 14% (n = 9/65) of the observations and a meeting with the OTLs ensued prior to community visits in only 3% (n = 2/65) of the observations.

OTLs and policy and program stakeholders attributed staff shortages to perceived high staff turnover due to career advancement and retirement.

*I think the solution is for them*, *those from the clinic*, *hiring enough staff to help them because when I* [got hired]*… they didn’t say I will go and help at the clinic and…*[also] *work with the Outreach Teams*. *They said you are an outreach team leader*. *They didn’t say you will work at the clinic*.

–OTL, IDI

*…we have tried to recruit young ones*, *the young ones now they want to upgrade their careers*. *They want to go to school*. *We try to hire the old ones*, *the old ones they work for few years and then they retire… so it’s a challenge*.

–Policy and program stakeholder, KII

#### Work environment

The work environment for the teams emerged as a third theme. Findings from both the qualitative and quantitative data showed that all participant cadres believed that the program was delivering high quality of care to communities and that CHWs and OTLs had high levels of job satisfaction. Reported challenges in the work environment included lack of supplies and material resources to support community-based activities.

*Perceived strengths*. All participant cadres noted that the surge in funding and support enabled scale-up of OT services in communities and that the teams had been successful in multiple domains: tracing and linking community members back to care; distributing medications in the community, thereby improving treatment adherence, increasing collaboration with other community organizations, and alleviating the burden on health facilities by reaching patients in their homes.

*…So*, *we use them* [OT] *for community awareness because with us we are mainly focused on the facility*, *but they are our eyes outside there in the community*. *They the ones that will go and see whatever is happening in the community*, *they will come and report back…they will take some of the services out there to the community*.

–Facility manager, IDI

Field observations showed a high rate of successful household visits by the teams. Of the 215 households visited during the 65 field-based observations, patients were located for 86% (n = 184/215) of the visits, enabling CHWs to provide services.

Another success was the high level of job satisfaction among CHWs and OTLs, many of whom viewed the teams as providing high-quality services to community members. CHWs noted that the teams had successfully re-connected many community members with health facilities and that home visits were making healthcare more easily accessible to community members.

*Household registration*, *we are good at that*. *We are good at tracing even though sometimes the patient*, *they give us the wrong address and telephone numbers*, *but tracing of TB and HIV patients*, *we are good at it*.

–OTL, IDI

*Our work is greater than any other*. *We have saved lives*, *eradicated illnesses*, *and avoided funerals in helping and nursing our community members back to health*. *When we arrived*, *the elders did not know how to consume their medication*. *Some overdosed*, *but now they do it diligently without us to monitor*.

–CHW, FGD

The positive feelings that OTLs and CHWs expressed about their work and contributions to improving community healthcare were affirmed in the KAP surveys: 84% (n = 161/191) of CHWs and 87% (n = 27/31) of OTLs rated the quality of the community-based care by the teams as “good” or “excellent” and 75% (n = 143/191) of CHWs and 84% (n = 26/31) of OTL were of the view that the services they provide meet the needs and expectations of the community. Additionally, 82% (n = 156/191) of CHWs and 84% (n = 26/31) of OTLs “strongly” or “somewhat” agreed with the statement, “if it were up to me, I would continue to work on the Outreach Teams for quite some time”.

**Fig 5:**
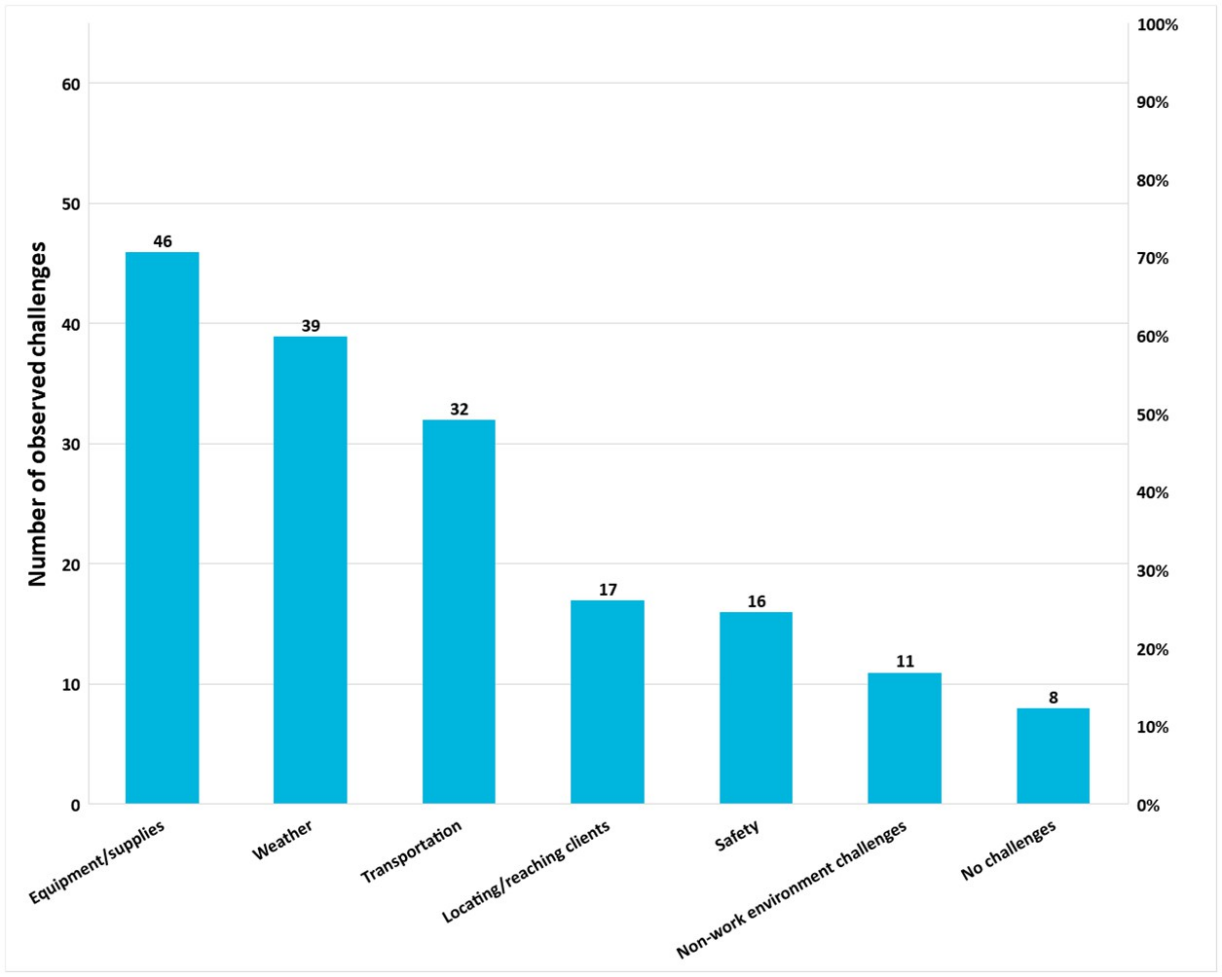
Observed work environment challenges for outreach teams, n = 65 field-based observations. *Categorization of observation challenges, other than ‘no challenges’, is not mutually exclusive.

*Perceived challenges*. Inadequate office equipment (printers, photocopiers), personal supplies (uniforms, umbrellas), weather conditions, lack of transportation, difficulty reaching and locating patients due to long distances between homes and incorrect contact information, and safety concerns were identified as key barriers in the work environment, as shown in Fig 5. All of these issues contributed to CHWs’ ability to work effectively in the community.

In 60% (n = 39/65) of field visits, CHWs were observed working under harsh weather conditions without protective supplies, such as umbrellas or sun hats. Some CHWs reported that they did not pack a lunch due to concern that it would spoil in the heat. Lack of adequate transportation for CHWs was observed in 49% (n = 32/65) of field visits. CHWs often walked long distances to reach their patients in excessive heat and without proper walking shoes. Travel time (walking) between the clinic and one or two households took over 30 minutes in 45% (n = 29/65) of observations. In the qualitative data, CHWs suggested that having uniforms would improve their safety by making them recognizable in the community as well as confer respect and credibility.

*We walk in the sun and in the rain without uniforms*. *We’ve been promised boots and raincoats*. *We’re getting old*, *about to go on the pension fund*, *and we’re still waiting for uniforms*. *The shoes we use are usually worn out by the end of the month*. *Some can’t even afford these R30 shoes*.

–CHW, FGD

Furthermore, in the IDIs, OTLs reported that they had to use their own transportation, which was costly, and thus impeded their ability to supervise CHWs on a frequent basis.

CHWs complained that they lacked supplies for monitoring patients’ health, such as sphygmomanometers and glucometers, and personal protective equipment when dealing with patients with a communicable disease, such as those with tuberculosis.

*If you are dealing with a high blood* [pressure] *patient*, *you need to keep checking their vitals*, *but without that machinery*, *it is pointless*.

–CHW, FGD

*You would sometimes be called to attend to a TB patient*, *but when you get to evaluate*, *you find that the TB has escalated to MDR stage*. *It is possible for it to spread…We don’t get shielded in any way*, *nor do we have the equipment to protect ourselves from those illnesses*. *They do not recognize us in that regard*. *We do not have benefits*.

–CHW, FGD

In 51% (n = 33/65) of the observations, study staff confirmed that CHWs did not have the supplies and monitoring equipment needed to provide some services to their patients, such as condoms, sphygmomanometers, HIV testing kits, gloves, masks, glucometers, and/or medication. CHWs also lacked the paper-based forms/tablets to register patients and collect patient data in 20% (n = 13/65) of the observations. Additionally, in the KAP surveys, only 52% (n = 99/191) of CHWs and 32% (n = 10/31) OTLs “strongly” or “somewhat agreed” that they consistently had the supplies they needed to perform their duties.

Another major challenge reported was an insufficient stipend. In the KAP surveys, only 42% (n = 13/31) of OTLs and 13% (n = 25/191) of CHWs reported satisfaction with their pay. This was also expressed in FGDs by CHWs who reported that they often had to use their own funds for photocopying, airtime, transportation, uniforms and weather-protective gear.

*…*[the] *weekly sheets that we use*, *they end up finishing*, *yes*, *they finish*. *The problem is that we struggle to get them when they finish*, *but we do go to the office to collect them but then we are asked to photocopy for ourselves*. *So at times even photocopying for yourself is expensive*, *they might charge you R2* [14 cents] *per page*, *and that delays the work that we* [are] *supposed to do*, *because of you not having enough material to work with*.

–CHW, FGD

A few participants reported that they had to pay for the repair or replacement of damaged work equipment, particularly the tablets they used for electronic data capturing. Many policy and program stakeholders acknowledged these funding challenges and expressed concerns about program sustainability given the lack of standardized compensation and payment scale for CHWs. They noted a disconnect between CHWs and their employers: although CHWs are on contracts, they wanted the same benefits as permanent employees.

*When programs are contract-based*, *you are appointed for two years and you’re not sure what’s gonna happen to you after two years… it creates a lot of uncertainty… we* [should] *stop appointing people short-term contracts*, *let’s appoint them on permanent contracts*. *Just like anybody else in the country*.

–Policy and program stakeholder, KII

Although research teams noted that patient homes were located and patients were reached in 86% (n = 184/215) of households visited during field observations, patient tracing was reported as a challenge for the teams. In the FGDs, CHWs reported that they are sometimes given incorrect addresses and/or the patients are not home when they arrive for the visit. This creates inefficiencies once in the field and causes delays in providing services in the community. CHWs also reported safety concerns, such as the fear of being physically or sexually attacked during household visits or on the street.

*We face problem such as being intimidated*. *For example*, *you’re carrying a schoolbag and it has pills or sometimes you are carrying a plastic and it’s visible that there’s pills inside*. *Then someone intimidates you and take*[s] *pills and injections since people sell injections too these days*.

–CHW, FGD

#### Monitoring and evaluation

The impact of the revised M&E strategy emerged as a fourth theme. Expanded M&E activities included new program indicators, data collection and database management processes, and mobile health (mHealth) tools and systems. Both updated M&E strategies and mHealth tools had only recently been introduced in the selected districts at the time of the evaluation.

*Perceived strengths*. Policy and program stakeholder KII participants and facility-level IDI participants noted that the introduction of the standardized indicators had the potential to enable more systematic data collection. Furthermore, use of mHealth enabled real-time and timely data collection, facilitated identification of households and follow-up visits, and has the potential to enhance supervision of CHWs, reduce data loss, improve data quality, and increase data confidentiality.

*It is working except that there are technical issues that we come across…but it’s really working and it reduced paperwork…and there is clean data…and confidentiality…now you don’t just see papers lying around*.

–Policy and program stakeholder, KII

*Perceived challenges*. Policy and program-level stakeholders indicated that it was unclear to them how OT data were synthesized and entered into the central database and then used to inform practice. They also noted shortcomings in the presentation of data summaries, particularly the inability to disaggregate results by district or facility. Additionally, the lack of dedicated data capturers at health facilities made it difficult to conduct routine M&E activities and reduced the potential of the revised M&E and mHealth tools to increase overall program efficiency.

*It’s just that it’s tough to break* [the data] *down…it’s for this clinic*, *it’s for that clinic…everything just goes in there…It’s not segregated*.

–Policy and program stakeholder, KII

*I feel*, *personally*, *if maybe they can give us more of trainings on that part*, *and also maybe we empower some of community healthcare workers to be at least the data capturers …because … again we don’t have data capturers*. *Those team leaders they have to be supervising and doing everything at the end of the month…So*, *at least*, *if we’ve got this M&E people who are like on daily basis doing such things*, *it will be better*.

–Policy and program stakeholder, KII

CHWs, in turn, felt that the phones and tablets that they used for data capturing made them vulnerable to theft and they lacked the necessary security to feel safe while traveling and visiting homes. Some CHWs commented that the amount and complexity of information to collect on the electronic forms prolonged household visits, which frustrated patients and caused tension. Connectivity problems caused the tablets and phones to freeze, further adding to patients’ and CHWs’ frustration.

*As for me*, *the problems that I have encountered started from the phone when we register*. *They welcome us well*, *but when you keep following the questions that are emerging from phones*, *they accuse us of taking time*. *There are many questions that are ask*[ed] *on the phones and they take time*. *You cannot just rush to the last question without answering the previous questions*. *The questions are many*, *hence people accuse us of wasting time*.

–CHW, FGD

In the FGDs, some CHWs highlighted the need for updated and functional servers and mHealth devices to preclude the need to revert to paper-based reporting. The research team observed low rates of both electronic and paper-based data entry during field visits: only in 9% (n = 6/65) of the visits were CHWs observed entering data electronically (a median of 47 minutes was spent on this activity; range = 15–67) and only in 17% (n = 11/65) were they observed using paper-based documentation (a median of 21 minutes was spent on this activity; range = 2–53). No sites were exclusively using electronic tools; twelve reported only using paper-based M&E data tools and the remaining eight sites reported using a combination of paper-based and electronic tools.

Some CHWs had lost their phones and tablets to thieves and had to pay out-of-pocket for replacements. CHWs also felt that the phones and tablets placed them under greater scrutiny from supervisors who could check up on them via Global Positioning System (GPS).

*They didn’t trust us*, *and we do our work*. *They don’t trust us still*. *That’s why they brought these phones that track our every movement*.

–CHW, FGD

## Discussion

Our findings highlight the complexities involved in strengthening and expanding a national public-sector CHW-based program. We provide insights into program components that are easier to implement versus those that are more complex and require longer-term and expanded investments. As we show in Fig 3, the four factors affecting program implementation—training, management, work environment and M&E—are intertwined and difficult to disentangle. Although training was delivered successfully to 1,664 OTLs and 22,216 CHWs between 2018–2019, with high rates of participant and stakeholder satisfaction and documented short-term improvements in CHW and OTL knowledge, the managerial and supervisory aspects of the program were more complicated, with challenges in the areas of ongoing training and mentorship, integration of the teams into health facility management structures, staffing shortages, and inadequate resource allocation to the teams. Despite these challenges, the overall sentiment toward the expanded program was positive across all participant cadres with notable successes, including hiring new staff, clarifying roles and responsibilities, and generally improved relationships between CHWs and OTLs. The early stages of implementing the more intensive M&E/mHealth approach also showed promise.

Many of the factors highlighted by our study–training, frequent and supportive supervision, ongoing mentorship, integrating CHWs with health facility-based providers, and financial and non-financial incentives to enhance CHW motivation–have been identified in the literature as key components of successful CHW programs [23–26]. CHW training programs have been shown to improve CHW knowledge and confidence levels [23]. We observed similar benefits, with CHWs and OTLs reporting that the intensive training increased their confidence in performing key tasks and had effectively prepared them for their roles. Both cadres demonstrated significant improvement in their test scores immediately following the training. The new training curriculum also helped to clarify roles, responsibilities, and supervisory structures, improving communication between CHWs and OTLs. Communication is a key component of supervision, and studies show that inadequate or poor supervision can negatively affect CHW motivation and performance [24, 25]. In the quantitative data in our study, CHWs reported they derived high levels of job satisfaction from serving the community and providing what they perceived to be good quality services. These findings affirmed those of a pilot study in seven sub-districts in the City of Tshwane, one of our study districts, which found a high level of overall satisfaction with training and roles and responsibilities among the majority of CHWs [26].

However, many studies caution that CHW training programs must be supported by regular refresher courses and ongoing supervision if their benefits are to be sustained [27, 28] because “classroom” skills and knowledge are not always directly transferable to practice and CHWs’ work in communities is typically strengthened by routine mentoring and support [29]. In our study we observed declines in CHW and OTL knowledge retention 3–12 months following the training, with some differences by district. Training of OTLs and CHWs was conducted in the same time frame in both districts by one non-governmental organization. Post-training support in each district was provided by two different PEPFAR implementing partners. However, we cannot say whether there were differential supportive supervision approaches, and if there were, whether this contributed to differences in post-training knowledge retention in the two districts. These differences highlight the critical importance of ongoing supportive supervision and in-service training. Many OTLs and CHWs in our study noted the need not only to extend the length of the initial training but to increase their skills and knowledge about health issues and policies through continuous in-service training and supervision. A study in rural Uganda found that CHWs who attended refresher trainings had more than 12 times the odds [aOR = 12.79 (95% CI: 1.02–159.26), p = 0.048] of higher task performance compared to those who had not received such training [30].

The ongoing supportive supervision and in-service refresher trainings planned for CHWs had not been fully implemented at the time of our study. Despite improvements in management and in relationships between OTLs and CHWs, we noted challenges with supervision attributable to staff shortages and other resource constraints. Some OTLs in our study were responsible for supervising as many as 50 CHWs and could not provide them with routine supportive supervision and mentorship. OTLs were also pulled into non-OT tasks at health facilities, which limited their availability for field-based supervision, as did their limited access to transportation. This was confirmed by field observations in which outreach teams were accompanied by their OTLs on only 12% of observed community visits and CHW teams almost never met with OTLs to plan and/or debrief before and after visits. This differs from the NDoH’s scope of work for OTLs, which states that OTLs are expected to spend 70% of their time in the field supporting CHWs during home visits and 30% time on administrative responsibilities at the health facility [12]. This likely contributed to the suboptimal knowledge retention in our study, as most CHWs lacked an opportunity for their OTLs to reinforce what they had learned in their didactic training in the field.

Supportive supervision is a continuous process that encompasses a range of activities, including on-the-job training, coaching and mentoring, performance feedback, strengthening relationships, building problem-identification and problem-solving skills, and working to improve resource allocation [31]. It has been shown to increase CHW motivation and has been associated with better performance, job satisfaction and quality of care [32, 33], suggesting that supportive supervision within OT and between health facility managers/staff and OTLs could improve OT performance [10]. However, a recent scoping review of non-physician primary healthcare workers in low- to middle-income countries found no consensus on the most effective approaches to supervision or the optimal intensity of supervision needed to improve service quality [34]. In a mixed-methods CHW supervision intervention study in four African countries, qualitative results indicated that supportive group supervision in combination with individual and/or peer supervision could improve CHW motivation and performance [35], but no significant changes were found in the quantitative results. Supervision that entailed problem-solving, skill-sharing and teamwork, and coaching was perceived to be especially supportive. A qualitative study of four cadres of CHWs in Eswatini reported that although additional training was perceived as one of the four changes that would likely lead to improved CHW performance, few participants viewed supervision and skills development for career progression as a key requirement [36].

Communication between facility managers and the teams in our study was not ideal and suggests that the program is not yet fully integrated into the health system. This is consistent with findings from an earlier South African study that reported strained relationships and lack of debriefings between CHWs and health facility staff [16, 17] and may be rooted in the historical origins of the program, when CHWs were managed by non-governmental organizations rather than the NDoH. A more recent study of 12 facility managers across three health districts participating in South Africa’s National Health Insurance pilot indicated that they were not trained or oriented on OTs at the beginning of the program and lacked understanding of their roles. In one district, facility managers received some training, but not until after the teams were operating in the communities [37]. The intended plan for facility manager training included a one-day training focused on the roles of the team members. In addition, facility managers noted the difficulty of attending to both teams and facility due to time constraints. In our study, facility managers and policy and program stakeholders highlighted the importance of the OTs in supporting community members in ways that health facility staff cannot. Nonetheless, despite this understanding, integration and acceptance of teams within health facilities were sub-optimal. This observation was echoed in a cross-sectional study of social and professional relationships in the OT system in the Ngaka Modiri Molema district of North West province [16]. Although supervision between OTLs and CHW in that study was satisfactory, health facility staff and middle managers had a limited role in supporting and monitoring the program. A recent study in South Africa’s Sedibeng district, Gauteng province, which explored CHW workplace trust, underscored the need for facility managers and nurses to mitigate social hierarchy in the facility so that CHWs would feel supported in their workplace [38].

Expanding the scope of training to include organizational relations, coordinating mechanisms, teamwork, and planned communication structures that allow for consistent dynamic interactions among all personnel involved in the OT initiative is one strategy to build organizational support for the program and for CHWs to feel the work they do is respected. However, a combination of non-financial and financial incentives is required, including supportive supervision to facilitate cohesion across all team members and other health facility staff, equity in pay, as well as respect and recognition. A review of 16 CHW programs in low- to middle-income countries noted that consistent management and supervision were the most frequently reported program design and management enabling factors for scale-up and sustainability, whereas insufficient incentives for CHWs, followed by weak management and supervision, were the most frequently cited barriers [39].

Policy and program stakeholders saw the revised M&E tools and the use of mHealth technology as an important innovation but acknowledged that the impact of these innovations was limited during the initial months of implementation, and that the new tools were not yet utilized to their fullest potential. In the early stages of implementation, the new M&E strategy also had some unintended consequences. Despite standardizing data collection, improving data quality and making data collection more efficient, in some instances tablet/smart phone-based data collection increased CHWs’ workload and may have contributed to reporting errors. Electronic data collection was challenged by network connectivity and hardware problems that slowed down data capture and made home visits too long and frustrating for patients and CHWs. CHWs also expressed concerns about their security when moving around in their communities with the tablets, which could be stolen, and about their work habits and performance being scrutinized by supervisors via GPS on their tablets.

Some of these issues have been corroborated in systematic reviews [40, 41]. A qualitative research synthesis review of 43 studies found that despite the potential for efficiency, healthcare workers’ real-world experiences were varied as to whether mHealth tools improved workflow, feedback and speed–they were considered efficient if these areas were enhanced, but inefficient if they slowed down work performance [40]. In a 2015 review of 140 studies of CHW programs, only 49 had an M&E system in place [24]. In a 2021 systematic review of mobile technologies for CHWs, based on 63 studies between 2009–2019 in 23 countries, only 6% assessed CHWs’ performance and adherence to clinical guidelines; 71% of studies utilized a health management information system/electronic medical record to standardize data entry and enable real time analysis of health problems within a community [42]. A 2019 scoping review of CHWs’ use of mHealth tools across continents and populations in 64 studies noted the dearth of reviews on CHWs and mHealth as well as lack of evaluation, experimental, and longitudinal studies [43].

Several evaluations of CHW programs in South Africa have used mHealth strategies to assess CHWs’ performance, although several reviews have not provided strong evidence to support their effectiveness [42, 44]. An evaluation in one sub-district in South Africa’s North West province compared the expediency, accuracy and supervisory proficiency of cell phone-based M&E to a paper-based M&E system and noted challenges with both [45]. Concerns about the paper-based system were related to carrying a bundle of papers in the field, confidentiality, and data accuracy. Compared to the mHealth system, only 40% of CHWs in that study showed a consistently high level of accuracy transferring weekly data to monthly data forms with paper-based monitoring; however, by the fifth month, discrepancies between the two systems were reduced, with all CHWs achieving a 90% or higher correspondence between phone and paper data [45]. An mHealth system can potentially improve the accuracy of M&E and enhance supervision of CHWs. In our study it was under-utilized; the paper-based system also documented few supervisory field visits.

mHealth applications are being increasingly used in low- to middle-income countries to facilitate work performance of CHWs and improve delivery of health services. mHealth M&E systems enable immediate data entry and thus real-time data monitoring and supportive supervision in the field. M&E systems are key to assessing the effectiveness of CHW programs. Although use of mHealth tools by CHWs has been increasing and the technology employed in mHealth has changed and evolved over time, there still are relatively few formal outcome evaluations and limited mHealth applications of CHW programs operating at scale in low- to middle-income countries [44].

Concerted and more intensive efforts are needed to improve CHWs’ comfort with expanded data collection and the use of mHealth technology to improve efficiency, quality and use of data for program improvement. Supervisors of frontline workers will need to be trained in mHealth data collection and see the benefits of this technology for OT health reporting systems. Regular debriefing meetings and review of daily logs, registers and weekly reports with supervisors to ensure that CHWs understand the meaning of and rationale for data categories collected could help to improve the quality of the data recorded by CHWs [41], as done in a study in Sedibeng health district, Gauteng province, South Africa [33]. Optimizing the M&E system is needed to ensure that CHWs can safely and reliably use mHealth tools and program managers can see relevant data disaggregated to additional levels of granularity (e.g., health facility). Comprehensive training, with continued provision of technical support, and supervision of CHWs’ data entry, verification and management, can help strengthen data accuracy and quality [46].

The effectiveness of the increased PEPFAR funding and NDoH support for the expanded program was compromised by shortages of critical equipment and supplies. CHWs in our study reported working without blood pressure machines, glucometers, or gloves, face masks, and other personal protective equipment. OTLs and CHWs alike complained about insufficient transportation support, which impeded regular supervision. These findings are consistent with earlier studies of CHWs in South Africa [33, 47–51]. Both CHWs and OTLs also expressed dissatisfaction with their salaries. Policy and program stakeholders acknowledged the funding gaps as a challenge and the CHWs’ and OTLs’ discontent with their salaries as a potential concern for program sustainability [51]. Resource challenges have been reported extensively by other studies of CHW programs in South Africa and other African countries [36, 47, 48, 52–54]. For example, the evaluation of a pilot project in seven sub-districts in the city of Tshwane found that nearly two-thirds of 431 CHWs were not satisfied with their monthly stipends [26] and another evaluation conducted in six South African provinces highlighted CHW’s low wages, insufficient numbers of CHWs and OTLs, and lack of uniforms and necessary equipment as barriers to program implementation. In addition, this study also reported that CHWs felt disrespected and lacked integration into the health facility with some mistrust noted among lower lay cadre staff members who feared that CHWs might replace them [55]. Several studies and reviews have indicated that both financial and non-financial incentives can increase job satisfaction and motivate improved task performance, and possibly retention, of CHWs [24, 56–59].

The issue of standardized CHW pay and benefits remains controversial and is further compounded by the effects of the global COVID-19 pandemic. CHWs in South Africa have carried out intermittent strike actions to protest non-standardized pay, benefits, and hours [60]. These continued and pervasive employment disputes likely undermine the efforts of NDoH and PEPFAR to strengthen OTs and their public health and HIV impact. Inconsistent remuneration of CHWs, including the general absence of formal benefits, has been a longstanding staffing challenge in the South African OT program. Prior to June 2018, there was no standardized CHW remuneration package; monthly salaries and stipends ranged nationally from ZAR1500-3000 (USD103–206). In 2018, the Public Health and Social Development Bargaining Counsel Resolution 1 set forth a public agreement to standardize compensation for CHWs with specified education or experience at ZAR 3500 (USD240) per month [61]. Implementation of this agreement was not uniform at the provincial level, and NDoH and PEPFAR interventions to date have not affected formal national adoption and allocated funding for standardized CHW remuneration.

Many of the challenges noted in earlier studies of CHW programs in South Africa and elsewhere in sub-Saharan Africa continue to be prevalent in South Africa’s OT program despite the 2018 surge of funding and technical assistance. These highlight the larger structural issue of sub-optimal integration of the OT into the public sector healthcare system. Smooth translation of health policy changes into efficient service delivery does not happen overnight, as it entails undoing routine operational practices of health systems and health facility staff and unlearning norms that militate against wholehearted acceptance of CHWs as essential workers. Providing CHWs with professional recognition, benefits and job security may be as critical to their integration into the health system as ongoing training and professional development activities.

### Study strengths and limitations

This study complements the growing literature on the OT program in South Africa and to our knowledge, is the only published study following the scale-up and revitalization brought about by new funding initiatives. With its focus on implementation of a specific “surge” of material and technical support provided by NDoH, PEPFAR and other stakeholders in 2018–2019, it contributes to our understanding of how the program works, and what is needed to enhance its performance. Analytic rigor was strengthened by triangulating the use of multiple quantitative and qualitative data collection methods from diverse stakeholders to enhance internal validity of evaluation results, in addition to large sample sizes for most data sources.

Several limitations of this evaluation must be noted. The evaluation was limited to 20 health facilities in two purposively-selected districts in two provinces and findings may not reflect the experience of OTLs and CHWs elsewhere in South Africa. While sample sizes were generally robust for the data collection methods, the online policymaker and program implementer survey sample, a supplemental data collection method to the KIIs, was small. The IDIs and KAP surveys were conducted in English. This may be a limitation due to language restrictions and potentially constrained generalizability. For the CHW and OTL knowledge test, the same CHWs and OTLs completed the I-TECH-administered pre- and post-test at the 20 participating sites in Bojanala and Tshwane; however, the repeat test administered with the KAP survey did not include all of the CHWs and OTLs who took the original pre- and post-test. The lack of repeat tests from all participants may have biased the results. By design, the study did not assess the perspective of patients or communities receiving OT services. Finally, as a formative process evaluation, the study assessed OT program implementation, not its impact, so we cannot determine whether the intensified support for OT improved health outcomes for beneficiaries.

## Conclusion

Given the prioritization of PHC and South Africa’s decentralization to a district health management system, the importance of CHWs as a bridge between the healthcare system and the community continues to grow. In addition to their role supporting health promotion and health education, OTs provide critical linkages to services for maternal and child health, tuberculosis, HIV, and other chronic conditions and are well poised to engage with emerging health issues such as the global COVID-19 pandemic [62]. Currently, CHWs in SSA [63, 64] are playing key roles in the response to COVID-19, including screening and referrals for testing, community education about mitigation strategies, promotion of vaccine uptake [65], and rectification of the rampant misinformation circulating in the public domain, and medication delivery [63, 64, 66, 67]. In the first month of the pandemic, 27,000 HIV and TB CHWs in South Africa were trained in COVID-19 screening who subsequently screened more than 11 million people (about 20% of the population) [68]. The 2018–2019 “surge” of support for OT programs brought critical resources to the task, enhanced and standardized training and oversight and introduced important M&E innovation. Additional attention to equipping OT with needed supplies and resources, ensuring proficient and continuous supportive supervision, building CHWs’ competencies, and enhancing professional and managerial relationships between the teams and health facilities will likely further improve OT performance and ensure that they continue to play a pivotal role in improving access to health services in the communities they serve.

## Supporting information

S1 File KII Guide—Policy and program stakeholders. (PDF)

S2 File IDI Guide—Facility-level staff. (PDF)

S3 File Focus group discussion guide. (PDF)

S4 File Online survey. (PDF)

S5 File KAP survey. (PDF)

S6 File Field-based observations/time motion guide. (PDF)

S7 File Site assessments tool. (PDF)

S1 Table Data collection strategy by sample and assessment domains. (PDF)
