# Supplementary material for: "They are our eyes outside there in the community": Implementing enhanced training, management and monitoring of South Africa’s ward-based primary healthcare outreach teams
Source: PLoS One. 2022 Aug 26;17(8):e0266445. doi: 10.1371/journal.pone.0266445 (PMC9417004; doi:10.1371/journal.pone.0266445)
Supplement: S1 File — (PDF) [file pone.0266445.s001.pdf]

Interview Guide for Central Level Informants

Part A – Implementation

INSTRUCTIONS TO INTERVIEWERS

Important information about using this interview guide:

1. Part A of this guide should be used only with Ward-Based Primary Healthcare Outreach Teams (WBPHCOT) partners *who are implementing activities related to WBPHCOT* (i.e. NDOH, district level DOH, WBPHCOT implementing partners, training partners, M&E partners). It should not be used with donors, or partners not directly engaged with implementation. Implementing partners should also complete Part B of the interview (see below).
2. ALL questions contained in this guide must be asked during the interview EXCEPT if stated otherwise.

Important information to give to respondents before conducting the interview:

1. Inform respondents that the aim of the process evaluation is to generate systematic, valid data from the expanded WBPHCOT activities to understand the implementation process and identify lessons learned.
2. Inform respondents that no names or personal identifiers will be included in transcripts or reports.
3. We estimate that the interview (Part A + Part B) will take approximately 60 minutes to complete.

Interview with: *(check one)*

O DOH at District Level (specify) _________________
O DOH at National Level
O DOH at Provincial Level (specify) _________________
O Implementing Partner (specify) ___________________________
O Other (specify) _______________________________

PLEASE CONFIRM THAT INFORMED CONSENT HAS BEEN OBTAINED AND DOCUMENTED BY INITIALING HERE:

___________________ (Interviewer's initials)

_____/ _____/ _____ (Date)

I. Description of WBPHCOT-Related Activities and Timeline

Question 1: When did [your organization] begin its work on the expanded WBPHCOT activities in [X location]?

[Probe for: first discussion, first planning activities, first implementation activities?]

Question 2: What WBPHCOT-related activities does [your organization] support?

[Ask respondent to describe WBPHCOT-related activities, quantifying whenever possible.]

Question 3: Does [your organization] include activities to increase the number or size of WBPHCOTs, e.g., support for staffing/hiring?

[If yes, ask respondent to describe activities, quantifying wherever possible. Examples may include hiring CHWs, hiring OTLs, adjusting staffing ratios or shifts, etc.]

Question 4: Does [your organization] include activities to improve the knowledge and skills of WBPHCOT staff, e.g., training, coaching, mentoring?

[If yes, ask respondent to describe activities, quantifying wherever possible.]

Question 5: Does [your organization] include activities to enhance documentation, monitoring and/or evaluation of WBPHCOT services? This would include routine M&E and mHealth, for example.

[If yes, ask respondent to describe M&E-activities, quantifying whenever possible. Examples may include support for developing M&E frameworks and tools, implementation of mHealth tools and resources, training staff to collect data using the tools, conducting data quality assurance activities, training staff to aggregate and analyze data, etc.]

Question 6: Does [your organization] include activities to enhance performance management of WBPHCOT staff?

[If yes, ask respondent to describe performance management activities. Examples may include support for job descriptions (setting standards), performance appraisal (assessing performance vs. standards), communication (e.g., systematic performance review) etc.]

Question 7: What elements of [your organization's] WBPHCOT support have been the most successful? The least successful? Why?

PROCEED TO PART B OF THE INTERVIEW

Part B – Lessons Learned

INSTRUCTIONS TO INTERVIEWERS

Important information about using this interview guide:

1. Part B starts with question #8.
2. Part B of this guide should be used with all WBPHCOT partners (i.e. National, District, and Provincial DOH, USG and other donors, civil society and WBPHCOT implementing partners).
3. *NB* that a subset of these respondents will also have been asked to complete Part A of the interview, and will be continuing straight to Part B.
4. ALL questions contained in this guide must be asked during the interview EXCEPT if stated otherwise.

Important information to give to respondents before conducting the interview:

1. Inform respondents that the aim of the process evaluation is to generate systematic, valid data from the expanded WBPHCOT activities to understand the implementation process and identify lessons learned.
2. Inform respondents that no names or personal identifiers will be included in transcripts or reports.
3. We estimate that the interview (Part B only) will take approximately 40 minutes to complete.

If respondent has not already completed Part A, please document: *(check one)*

O DOH at District Level (specify) _________________
O DOH at Provincial Level (specify) _________________
O DOH at National Level
O Implementing Partner (specify) _________________
O Civil Society (specify) _____________
O Academic Partner (specify) ________________
O Donor (specify) _________________________
O Other (specify) _______________________________

PLEASE CONFIRM THAT INFORMED CONSENT HAS BEEN OBTAINED AND DOCUMENTED BY INITIALING HERE:

__________________ (Interviewer's initials)

II. Implementation Successes and Challenges

Question 8: To what extent have the expanded WBPHCOT activities been implemented in [location]? Has support for ward-based PHC outreach teams been delivered as planned?

[Probe for concrete examples of activities that were – or were not – implemented, in the domains of training, staffing, M&E, mHealth and management.]

Question 8a: To follow up on the previous question, on a scale of 1 to 10, where 1 is *not implemented at all*, and 10 is *completely implemented*, to what extent have the expanded WBPHCOT training activities been implemented as planned?

[Once a number has been given, remind the informant of the scale, characterize the response, and confirm the choice of number.]

Question 8b: On a scale of 1 to 10, where 1 is not implemented at all, and 10 is completely implemented, to what extent have the expanded WBPHCOT staffing activities been implemented as planned?

[Once a number has been given, remind the informant of the scale, characterize the response, and confirm the choice of number.]

Question 8c: On a scale of 1 to 10, where 1 is not implemented at all, and 10 is completely implemented, to what extent have the expanded WBPHCOT M&E activities been implemented as planned?

[Once a number has been given, remind the informant of the scale, characterize the response, and confirm the choice of number.]

Question 8d: On a scale of 1 to 10, where 1 is not implemented at all, and 10 is completely implemented, to what extent have the expanded WBPHCOT mHealth activities been implemented as planned?

[Once a number has been given, remind the informant of the scale, characterize the response, and confirm the choice of number.]

Question 8e: On a scale of 1 to 10, where 1 is not implemented at all, and 10 is completely implemented, to what extent have the expanded WBPHCOT management activities been implemented as planned?

[Once a number has been given, remind the informant of the scale, characterize the response, and confirm the choice of number.]

Question 9: On a scale of 1 to 10, where 1 is not well at all, and 10 is very well, how do you think the expanded WBPHCOT activities in [location] are working, overall?

[Once a number has been given, remind the informant of the scale, characterize the response, and confirm the choice of number.]

Question 9a: To follow up on the previous question, on a scale of 1 to 10, where 1 is *not working well at all*, and 10 is *working very well*, how do you think the expanded WBPHCOT training activities are working?

[Once a number has been given, remind the informant of the scale, characterize the response, and confirm the choice of number.]

Question 9b: On a scale of 1 to 10, where 1 is not working well at all, and 10 is working very well, how do you think the expanded WBPHCOT staffing activities are working?

[Once a number has been given, remind the informant of the scale, characterize the response, and confirm the choice of number.]

Question 9c: On a scale of 1 to 10, where 1 is not working well at all, and 10 is working very well, how do you think the expanded WBPHCOT M&E activities are working?

[Once a number has been given, remind the informant of the scale, characterize the response, and confirm the choice of number.]

Question 9d: On a scale of 1 to 10, where 1 is not working well at all, and 10 is working very well, how do you think the expanded WBPHCOT mHealth activities are working?

[Once a number has been given, remind the informant of the scale, characterize the response, and confirm the choice of number.]

Question 9e: On a scale of 1 to 10, where 1 is not working well at all, and 10 is working very well, how do you think the expanded WBPHCOT management activities are working?

[Once a number has been given, remind the informant of the scale, characterize the response, and confirm the choice of number.]

Question 10: What are the most important successes of the expanded WBPHCOT activities to date and why?

[Probe for concrete examples of positive activities, outputs or outcomes.]

Question 11: What are the three main factors that have facilitated the implementation of the expanded WBPHCOT activities?

[Probe for concrete examples of people, systems, environments, etc. that facilitated implementation. Make sure to get three examples]

Question 12: What are the three main challenges to the implementation of the expanded WBPHCOT activities?

[Probe for concrete examples of people, systems, environments, etc. that hindered implementation. Make sure to get three examples]

Question 13: Have any changes or modifications been made to the expanded WBPHCOT activities since they were initially planned? Why were these changes made?

[Probe for specific examples, if any.]

Question 14: Are there innovations—activities, ways of working, service delivery, other—that you have observed during the implementation of the expanded WBPHCOT activities?

[Probe for specific examples, if any.]

III. Results and Unintended Consequences

Question 15: What impact would you say the expanded WBPHCOT activities have had so far?

[Probe beyond anecdotes – are there concrete data?]

Question 16: What unintended consequences, either positive or negative, have you observed during implementation of the expanded WBPHCOT activities?

IV. Best Practices

Question 17: Based on your overall experiences with the expanded WBPHCOT activities to date, what aspects of the expansion do you feel have worked well and should stay the same as the program moves forward?

[What are best practices that should be supported as the expanded WBPHCOT activities roll out in new districts and sub-districts?]

Question 18: Based on your overall experiences with the expanded WBPHCOT activities to date, what aspects of the expansion do you feel have not worked well and should be changed as the program moves forward?

[What should be changed as the expanded WBPHCOT activities roll out in new districts and sub-districts?]

Question 19: On a scale of one to ten, where 1 is poor and 10 is excellent, how would you describe the quality of the expanded WBPHCOT activities to date based on the information you currently have?

[Once a number has been given, remind the informant of the scale, characterize the response, and confirm the choice of number.]

a. [Ask only if the response above is < 10]: How did you reach this conclusion? What can be done to improve the quality of expanded WBPHCOT activities?

Question 20: Is there anything else should we know about the expanded WBPHCOT activities that has not been covered in this interview?

Thank you for your time.
