# Supplementary material for: "They are our eyes outside there in the community": Implementing enhanced training, management and monitoring of South Africa’s ward-based primary healthcare outreach teams
Source: PLoS One. 2022 Aug 26;17(8):e0266445. doi: 10.1371/journal.pone.0266445 (PMC9417004; doi:10.1371/journal.pone.0266445)
Supplement: S2 File — (PDF) [file pone.0266445.s002.pdf]

Interview Guide for Site-Level Informants

INSTRUCTIONS TO INTERVIEWERS

Important information about using this interview guide:

1. This guide should be used with health facility managers, outreach team leaders (OTLs) supervising Ward-Based Primary Healthcare Outreach Teams (WBPHCOT), and other health facility staff working directly with WBPHCOT teams and/or clients referred by WBPHCOTs to the facility (e.g., lay counselors, others). It should not be used with site-level staff who are not directly engaged with the WBPHCOT teams.
2. ALL questions contained in this guide must be asked during the interview EXCEPT if stated otherwise.

Important information to give to participants before conducting the interview:

1. Inform participants that the aim of the process evaluation is to generate systematic, valid data from the expanded WBPHCOT activities to understand the implementation process and identify lessons learned.
2. Note that the guide has a mix of open-ended and close-ended questions.
3. Inform participants that no names or personal identifiers will be included in transcripts or reports.
4. We estimate that the interview will take approximately 60 minutes to complete.

Interview with: *(check one)*

O Facility Manager
O OTL
O Other clinician (doctor, nurse, medical officer)
O Other non-clinical staff (lay counselors, data clerk, pharmacy staff) – *specify*___________________
O Other *specify*____

READ CONSENT FORM and obtain consent from participant before you start the interview. After beginning the recorder, verify verbally that interviewee consents to audio-recording.:

Interviewer Initials: __________

Date: _____/ _____/ _____

Location/Facility Code: __________________

A. Experience and Training [all cadres of participants]

I would like to start by asking a few questions about your professional background.

1. For how many years have you been working in this district?
2. What is your role at this facility? What are your main responsibilities?
3. For how many years have you been working at this facility?
4. How does your job relate to the WBPHCOT team(s) at this facility?

B. Facility and Context [all cadres of participants]

I would like to learn a little bit more about this facility and community.

5. What are the top three general problems facing the community served by this health facility? *[This could be social problems, economic problems, health problems, or other – the question is intended to be open-ended]*
6. What are the top three leading causes of death and disability – for the community served by this health facility?
7. Thinking about HIV services, what is needed to improve HIV testing services in this community? How could more people living with HIV be reached, tested, and linked to treatment?
8. What about HIV treatment services in this community -- In what ways could services be improved to increase the number of people who are retained in care and on treatment?

C. Description of the Expanded WBPHCOT Activities [Facility Managers and OTLs only]

I would like to learn more about the ward-based outreach teams.

For Facility Managers ONLY:

9. What is the role/function of the WBPHCOT team at this facility?

10. Who supervises the WBPHCOT team?

11. You may have heard about the new WBPHCOT activities that were expanded earlier this year? Could you describe what it involves?
__PROBE for__ revised CHW scope of work, revised M&E forms, revised training, enhanced staffing, other.

Staffing & Training

12. I would like to ask you about some recent changes in the WBPHCOT program:
    a. The scope of work for community health workers (CHWs) on WBPHCOT teams has recently been updated – have you seen the final version? *[show example]*
    O Yes O No

    b. The training curriculum for WBPHCOT staff has recently been revised – are you familiar with the content of the latest training?
    O Yes O No

M&E & mHealth

c. The Management and Evaluation (M&E) framework and tools used by CHWs and OTLs has recently been updated – are you familiar with the latest data collection tools? *[show example]*
   O Yes   O No

d. Are there mHealth tools for WBPHCOT in use at this facility?
   O Yes   O No

Now SKIP to Question 26

For OTLs ONLY:

13. When did you start your job as an OTL?

Training

14. Can you tell me about your training to be an OTL?

    PROBE for *off-site training vs. on-site training, didactic training vs. supportive supervision, other.*

15. Have you received any training on the WBPHCOT program in the past six months?
    a. Please describe (when/where/how many days)
    b. On what elements of the program were you trained?

       PROBE for *management, M&E, technical (e.g., linkage, defaulter tracking.*
    c. How effective was the training? How equipped do you feel for your role on the WBPHCOT team?

Management

16. Can you describe your exact role and responsibilities as the leader of the outreach team? Tell me about what you do in a typical week? In a typical month?
17. How much (approximately what percent) of your time is spent supervising the outreach team?

    If answer is 100%, SKIP to question 18.

    17a: What are your other responsibilities, e.g., what else do you do apart from supervising the outreach team?

    17b. Do you have sufficient time for OTL supervision and these other responsibilities?
18. How does the outreach team decide which households will be visited in a given week?
19. What are some of the common issues that arise from household visits, and how does the outreach team usually handle them?
20. How often does the team meet to discuss the household visits? What is your role in these meetings?
21. In a typical week, how often do you leave the health facility and go to the community with the outreach team? What about in a typical month?

22. In your experience, how would you describe the relationship between outreach team leaders and CHWs? Is it generally collegial, or are there challenges?
23. Do you receive any ongoing mentoring or supportive supervision to assist you to lead the outreach team? Please describe.
24. What are your challenges to supervising and managing the WBPCHOT? How often (% time) are you re-assigned to other duties? Please describe how this happens, the duties you are re-assigned to and what happens to the WBPHCOT on these days? What would you see as solutions to these issues?
25. What are other barriers you face in successfully supervising the WBPHCOT? ***PROBE for*** *Challenges with personnel, policies, resources, training, salaries, etc.* What do you see as solutions to these issues?

For BOTH OTLs and Facility Managers:

Management

26. How do you determine if the WBPHCOT team at this facility is doing a good job?
Probe for *existence of standards, achievement of targets, client feedback, quality of work, other*.

27. I have a few additional questions about WBPHCOT supervision.
    a. Do the staff of the WBPHCOT teams (OTL, CHWs) have formal job descriptions?
    O Yes O No O Don't know

    IF YES, can you share them with me?

    b. Do the staff of the WBPHCOT teams (OTL, CHWs) receive formal performance reviews?
    O Yes O No O Don't know

    IF YES, how frequently? Are those documented? Can you share an example of a blank performance review form with me?

    c. Do the staff of the WBPHCOT teams (OTL, CHWs) receive regular feedback about their work?
    O Yes O No O Don't know

    IF YES, what standards are used to determine if individuals are doing a good job? ***PROBE for*** *volume of services, professionalism, knowledge, skills, other*

    d. How effective is the supervision of the WBPHCOT, in general? *Please choose one best answer*:
        O Not effective, could be improved
        O Somewhat effective, works fairly well
        O Very effective, no need for improvement

Staffing

28. When was the new I-TECH WBPHCOT curriculum adopted by the site?
29. Have there been any staffing changes in the WBPHCOT team since [DATE from #28]? IF YES, please describe.
30. Has the WBPHCOT team received any training since [DATE from #28]? IF YES, please describe.
31. Have there been any changes in the way the WBPHCOT team collects and uses data since [DATE from #28]? IF YES, please describe.
32. Have there been any changes in the way the WBPHCOT team is managed/supervised since [DATE from #28]? IF YES, please describe.
33. Is the size of the WBPHCOT team(s) at this facility appropriate for their workload?
    - O Yes, about right
    - O No, too large
    - O No, too small

D. Perceptions of the WBPHCOT [all cadres of participants]

I would like to ask you to reflect on your experience with the outreach team

33. On a scale of 1 to 10, where 1 *is not effective at all*, and 10 is *very effective*, how would you rate the overall effectiveness of the WBPHCOTs in this community/ward?

    [Once a number has been given, remind the person of the scale, characterize the response, and confirm the choice of number.]

34. Which aspects of the outreach team do you consider to have been successful? ***PROBE*** *positive elements*. What are the team's three biggest achievements?

35. Which aspects of the outreach team do you consider to be less than successful? ***PROBE*** *negative elements*. Where has the team fallen short?

36. What would you say are the three most important barriers to success? ***for***

37. What would you say are the three top solutions to these barriers? ***for***

E. Impact of WBPHCOT in the community [all cadres of participants]

I would like to ask about any changes that may have occurred due to the WBPHCOT program, with a focus on HIV services

<u>On a scale of one to 10, where 1 is *No Impact* and 10 is *Major Impact*:</u>

39. To what extent do you think the WBPHCOT program has increased the *demand* for (or interest in) HIV testing services in this community?

    (1-No impact; 10-Major impact).

40. To what extent have WBPHCOT activities addressed *uptake* of HIV testing services in this community (meaning community members that actually get tested for HIV

    (1-No impact; 10-Major impact).

41. To what extent do you think the WBPHCOT program has been able to increase linkage to treatment/ART for clients who test positive for HIV (meaning persons who test positive for HIV seek and receive care from a medical facility)?

    (1-No impact; 10-Major impact).

41. To what extent do you think the WBPHCOT program has been able to improve adherence to ART medications for clients being treated for HIV (meaning start and stay on HIV treatment)?

    a. *(1-No impact; 10-Major impact).*

42. To what extent do you think the WBPHCOT program has been able to improve retention in care for patients on ART, for example by conducting defaulter tracing (meaning sustaining HIV treatment)?

    a. *(1-No impact; 10-Major impact).*

43. To what extent do you think the WBPHCOT program has been able to improve defaulter tracing?

    (1-No impact; 10-Major impact).

F. Best practices, Next Steps for WBPHCOT program **[all cadres of participants]**

Thinking about the future of the WBPHCOT program:

44. Which parts of the WBPHCOT program should stay the same as the program grows and expands to other sub-districts?
45. Which parts of the program should be changed as the program grows and expands to other districts?
46. What else would you like us to know about your work and the WBPHCOT program that we have not covered in this interview?

Thank you for your time.
