# Supplementary material for: "They are our eyes outside there in the community": Implementing enhanced training, management and monitoring of South Africa’s ward-based primary healthcare outreach teams
Source: PLoS One. 2022 Aug 26;17(8):e0266445. doi: 10.1371/journal.pone.0266445 (PMC9417004; doi:10.1371/journal.pone.0266445)
Supplement: S3 File — (PDF) [file pone.0266445.s003.pdf]

Focus Group Discussion Guide for Community Health Workers

FOCUS GROUP DISCUSSION (FGD) GUIDE	
FGD Identification Number	
FGD Facilitator Name	
FGD Note Taker	
Date of FGD	
FGD Site ID Number	
# of FGD Participants	
FGD Start Time (HH:MM)	
FGD Stop Time (HH:MM)	
Data Check Performed by	
Data Transcription Date	
Transcribed by	

Before we start, I want to remind you of basic guidelines which we should all observe:

- We are interested in your personal experiences and opinions. There are no right or wrong answers.
- If there are any questions that you feel uncomfortable answering, you may skip those.
- Everything we talk about is confidential.
- During the discussion, we ask that you not use real names or anything that would identify others.
- We ask each participant to keep what is said in this group to himself/herself and not gossip about other people's contributions.
- The information gathered will not affect or interfere with your employment or performance reviews in any way.
- The focus group discussion will be audio-recorded to ensure that it is carried out as planned, and to help us in supplementing our written notes and ensuring their accuracy. The recording will only begin after introductions are complete.
- The focus group discussion will last approximately 1.5 hours.

Focus Group Discussion Guide for Community Health Workers

Field Notes

A. Introductions

1. Let us go around the room: please introduce yourself and tell us a little bit about yourself.

2. How long have you been working as a community health worker?

3. How long have you been working on a WBPHCOT team?

[Turn on audio recorder here and inform participants that recording has begun.]

B. Context

4. What are the main health challenges facing the community where you work?

5. What are the main challenges related to HIV facing the community where you work?

6. What makes it easy or hard for people in the community where you work to access HIV testing services?

7. What makes it easy or hard for people in your community to start antiretroviral treatment (ART) when they test positive for HIV?

8. What makes it easy or hard for people on ART in your community to take their medicine regularly ("every dose, every day")?

9. What makes it easy or hard for people on ART in your community to remain in care and attend all their appointments?

C. WBPHCOT Staffing and Characteristics

10. What are the day-to-day activities of the outreach team? Can you tell me about what you do on a typical day? A typical week? ***PROBE for*** *time spent in community vs. facility, and time spent in transit vs. with clients. Also* ***PROBE for*** *type of health issues addressed, e.g., MNCH, HIV, TB, NCDs, mental health*

Field Notes

11. What are the most challenging/frustrating things that you face on the outreach team?

[Capture these variables on a flip chart, ensure that everyone has contributed and that you have all the ideas about the most challenging/frustrating activities. Then ask the participants to "vote" for the top three. Document by photographing the flip chart, making sure not to include images of any participants.]

12. What are the most rewarding/gratifying things that you do on the outreach team?

[Capture these variables on a flip chart, ensure that everyone has contributed and that you have all the ideas about the most rewarding/gratifying activities. Then ask the participants to "vote" for the top three. Document by photographing the flip chart, making sure not to include images of any participants.]

D. Preparation, Training, Supervision and Support

13. What type of training do CHWs receive to support their work on outreach teams?

14. When were you last trained on outreach team activities? Could you tell us more about this most recent training? **PROBE for** whether it was formal didactic training or supportive supervision, whether it was off-site or at the health facility.

15. What do you think about CHW training for outreach team activities? **PROBE for** whether Ps think training is appropriate and sufficient, whether more or different training would be better.

16. Are there other things that you would like to learn in order to improve your performance as an outreach team CHW? ***PROBE for*** *concerns about lack of knowledge or skills in specific areas.*

17. What cadre of health worker supervises the outreach team? To the best of your knowledge, what are the supervisor/team leader's qualifications? ***PROBE for*** *concerns about outreach team leader experience and/or training.*

18. How often do supervisors (outreach team leaders) meet with the team? Where do they meet with the team? Do the supervisors come to the community with the team? What

Field Notes

inputs or feedback do they provide to the CHW team? ***PROBE for*** *feedback about quantity of services, professionalism, knowledge and practice, and growth/improvement.*

19. Do you think this level and style of supervision is appropriate and sufficient, or would you prefer a different approach? ***PROBE for*** *specifics, including relationships between OTL and CHWs; what would make supervision better.*

20. What transportation, equipment and supplies do CHW receive to assist them with their work? ***PROBE*** *for challenges.*

E. Performance Management

21. How were you oriented to your roles and responsibilities on the outreach team? Do you have a formal written job description?

22. How do you know if your outreach team (as a whole) is doing a good job? ***PROBE for*** *feedback from OTLs; review of data on quantity of services; review of data on quality of services; feedback from clients, other.*

23. How do you know if you as an individual CHW are doing a good job?

24. What are the standards for individual CHW performance on outreach teams? What would a CHW on a WBPHCOT have to do in order to get a great performance review? **PROBE for** whether given a written copy of these standards, informed verbally about them, or not informed at all.

25. Do you receive feedback on your performance? Who provides feedback and how frequently? ***PROBE for*** *feedback on professionalism, volume of services, quality of services, quality of documentation, other.*

26. Overall, how would you rate the quality of the services you (as an individual) and your team (as a group) provide to clients in your community? ***PROBE for*** *what is working well and what could be better.*

F. Documentation and M&E

Field Notes

Now I would like to change topics and ask you about the tools you use in your outreach work.

28.
   (a) What forms or tools do you use to plan or prepare for outreach visits and/or follow up on things from previous outreach visits? ***PROBE for*** *Diary, Daily Activity Sheet, Referral Form, Household Record, and Tracing Form.*

   (b) How is the information on these forms or tools used?

29.
   (a) What forms or tools do you use as part of outreach visits? ***PROBE for*** *Daily Activity Sheet, Referral form, Household Record, and Tracing form.*

   (b) How is the information you collect used?

30. Do you feel comfortable using these tools? Was your training sufficient?

31. Do you complete weekly and monthly summary sheets? Is this process fairly simple, or are there difficult aspects?

32. Do you use any electronic or mobile tools to document WBPHCOT activities? IF YES, could you describe those? *IF NO, SKIP TO SECTION G.*

33. How easy or hard is it to use the electronic/mobile tools? Why?

34. Do the electronic/mobile tools save you time?

35. What are the advantages and disadvantages of using these electronic/mobile tools?

G. Impact of WBPHCOTs in the Community

Now I want to ask you about the effect you think that the WBPHCOT has had in your community.

36. How would you rate the impact of the outreach teams in this community? *Rate on a scale of 1-10 from 1 = worst to 10 = best].* Please explain why.

Field Notes

37. Have outreach team activities led to more visits to the health facility?

    O If consensus is YES → probe for why this has happened, and who is coming to the clinic that would not have come before the outreach team started work

    O If consensus is NO → probe for why this has not happened

38. Have outreach team activities led to more HIV testing and treatment?

    O If consensus is YES → probe for why this has happened, and who is being tested that would not have been before the outreach team started work

    O If consensus is NO → probe for why this has not happened

39. What do you consider to be the main successes of the WBPHCOT program in your community? Please explain.

40. What do you consider to be the main challenges facing the WBPHCOT program in your community? Please explain.

H. Barriers, Next Steps

41. What do you consider to be the three most important barriers to HIV testing, treatment and retention in this community that the WBPHCOT program has not addressed/addressed enough?

42. What do the outreach teams need to expand their successes?

43. If you were given 2000 rand, and asked to improve the program for patients in your community, what changes would you suggest?

44. What else would you like us to know that we have not covered in this interview?

Field Notes

Thank you.
