# Supplementary material for: "They are our eyes outside there in the community": Implementing enhanced training, management and monitoring of South Africa’s ward-based primary healthcare outreach teams
Source: PLoS One. 2022 Aug 26;17(8):e0266445. doi: 10.1371/journal.pone.0266445 (PMC9417004; doi:10.1371/journal.pone.0266445)
Supplement: S4 File — (PDF) [file pone.0266445.s004.pdf]

Online WBPHCOT Partners

Flesh-Kincaid Reading level = 6.7

CONSENT

Study Title:
Strengthening Ward-Based Primary Healthcare Outreach Teams (WBPHCOT) to Support HIV Epidemic Control: A Process Evaluation of the Expanded WBPHCOT Activities in South Africa
Investigators:
ICAP-Columbia University: Dr. Miriam Rabkin, Ms. Blanche Pitt
Institution:
ICAP-Columbia University, New York, NY USA

Introduction:
In collaboration with the US Centers for Disease Control and Prevention (CDC) and the National Department of Health (NDOH) in South Africa, ICAP at Columbia University in New York, USA, is conducting an evaluation of the Ward-Based Primary Healthcare Outreach Teams (WBPHCOT) program in South Africa.

You are being invited to take part in an online questionnaire because we want to learn more about the design, development and implementation of the expanded WBPHCOT activities. Findings from this evaluation will be used to inform ongoing implementation by providing insights into the successes, challenges, and potential areas for improvement of the expanded WBPHCOT activities.

What do I have to do if I agree to take part?
If you agree to take part, the questionnaire will take about 30 minutes. We will ask you both closed- and open-ended questions. You will be asked to share your thoughts and opinions on topics, including outreach team training, remuneration and staffing; the PEPFAR surge; WBPHCOT activities implemented; lessons learned; successes and challenges; areas for improvement; and views on best practices and next steps.

We seek your honest answers. There are no right or wrong answers. All your questions will remain confidential and your name will never be linked to any of your responses.

What are the potential risks?
Some of the questions may make you uncomfortable. You do not have to answer any questions that may make you uncomfortable.

We have developed procedures to protect your confidentiality. However, there is always a very small chance of loss of confidentiality. To avoid this, all staff working on this study will be trained on participant confidentiality and your name will not appear anywhere with your responses. Other procedures for protecting your confidentiality are described below.

What about confidentiality?
This program evaluation has been approved by the Columbia University Institutional Review Board and the Human Sciences Research Council (HSRC), South Africa.

We will do everything we can to keep your information confidential. Your name will not be written on any forms and will not be used in connection with anything that you tell us. Only the following individuals and/or agencies will be able to look at the data that are collected:

- The investigators and study staff
- Authorities from Columbia University including the Institutional Review Board (IRB)
- The Human Sciences Research Council Research Ethics Committee (HSRC REC)
- The US Centers for Disease Control and Prevention (CDC)

No identifying information will be shared with anyone outside of the people listed above. Identifiers will be removed from the information collected. Your permission allows us to use and share de-identified information we collect for future research studies without additional consent and with no time limit.

Your responses will be collected and stored on a secure password-protected server and only study staff will have access to this.

Are there any costs?
You will not pay for any costs related to the questionnaire.

Will I be given anything for taking part?
You will not receive any compensation for taking part in this questionnaire.

Can I decide not to complete the questionnaire?
You are free to stop participating at any time or refuse to take part in any or all parts of this questionnaire. If you choose to not take part or complete the questionnaire, it will not affect you in any way.

Questions/Points of contact:
If you have any questions about the questionnaire or feel that you have been harmed by taking part, you should contact the Co-Investigator, Ms. Blanche Pitt, who can be reached at:

Blanche Pitt, MSc
Co-Investigator
Country Director, ICAP South Africa
446 Rigel Avenue
Rigel Park, Block A
Unit 202AS, Erasmurand
Pretoria 0181, South Africa
Telephone: +27 12 360 0640
Email: bp2360@cumc.columbia.edu

If you have any questions about your rights as a participant in this study, please contact the

Columbia University Institutional Review Board
Telephone: 011 212-305-5883 Email: irboffice@columbia.edu

Human Sciences Research Council (HSRC):
Telephone: +27 12 302 2000

Online Consent:
Do you have any questions about the questionnaire? If so, please contact Blanche Pitt at +27 12 360 0640.
Study participant's statement:

This study has been explained to me. I volunteer to take part in this study. I have had a chance to ask questions. If I have questions later about the study, or if I have been harmed by participating in this study, I can contact Blanche Pitt, listed on the first page of this consent form. If I have questions about my rights as a research subject, I can call the Columbia Unversity Institutional Review Baord at 011 212-305-5883 or the Human Sciences Research Countril (HSRC) at +27 12 302 2000. I have printed a copy of this consent form for my records.

_____ I agree

_____ I decline

INSTRUCTIONS
This electronic questionnaire is part of a process evaluation of WBPHCOT intended to generate systematic, valid data to support further implementation of revitalized WBPHCOT support activities. This anonymous questionnaire has a mix of open-ended and close-ended questions, and should take approximately 30 minutes to complete. No names or personal identifiers will be collected.

Please indicate the institution you represent: *(check one)*

O National DOH
O Provincial DOH
O Regional Training Center
O Implementing Partner (*please specify*) ________________
O Civil Society (*please specify*) ____________
O Other (*please specify*) ____________________________

A. Implementation Successes and Challenges

These questions assess the extent to which the expanded WBPHCOT activities have been implemented as planned. By expanded activities, we mean implementation of: (1) enhanced staffing of WBPCHOTs; (2) updated training for WBPHCOT community health workers (CHWs) and outreach team leaders (OTL); (3) improved management and oversight of WBPHCOT CHW teams; (4) implementation of new monitoring and evaluation tools for WBPCHOTs; and (5) any additional activities planned/implemented.

Coverage, M&E, & mHealth

A1a. At how many health facilities did [your province/district/organization] plan to implement the expanded WBPHCOT activities? ________ □ Don't know (*tick*)

A1b. To date, how many of these facilities have implemented the expanded WBPHCOT activities? ________ □ Don't know (*tick*)

A1c. To date, how many facilities are using updated monitoring and evaluation tools to document the expanded WBPHCOT activities? ________ □ Don't know (*tick*)

A1d. To date, how many facilities are using mHealth tools to document the expanded WBPHCOT activities? ________ □ Don't know (*tick*)

Staffing

A2a. Did [your province/district/organization] plan to hire new CHWs specifically to support the expanded WBPHCOT activities? IF YES, how many? ________ □ Don't know (*tick*)

A2b. To date, how many CHW have been hired specifically to support the expanded WBPCHOT activities? ________ □ Don't know (*tick*)

A2c. Did [your province/district/organization] plan to hire new outreach team leaders (OTLs) to support the expanded WBPHCOT activities? IF YES, how many? ________ □ Don't know (*tick*)

A2d. To date, how many OTLs have been hired to support the expanded WBPCOT activities? ________ □ Don't know (*tick*)

Training/Management

A3a. How many CHWs did [your province/district/organization] plan to train for the expanded WBPHCOT activities? _______ □ Don't know (*tick*)

A3b. To date, how many CHW have been trained? ________ □ Don't know (*tick*)

A3c. How many OTLs did [your province/district/organization] plan to train for the expanded WBPHCOT activities? _______ □ Don't know (*tick*)

A3d. To date, how many OTLs have been trained? ________ □ Don't know (*tick*)

A3e. Which training curriculum was used to train CHWs to support WBPHCOT?

_____ Curriculum developed by I-TECH and partners and launched in mid-2018?

_____ Other (*please specify*)____

_____ Don't know

_____ N/A

Overall Implementation

A4. On a scale of 1 to 10, where 1 is *not well at all*, and 10 is *very well*, how well do you think the expanded WBPHCOT activities in your province is working? ________ □ Don't know (*tick*)

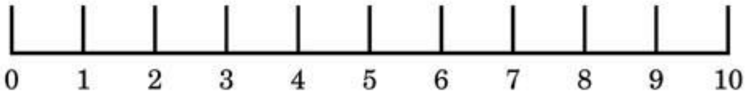

A5. What are the most important successes of the expanded WBPHCOT activities to date? *(Select all important successes)*

_____ Training of CHW and OTLs

_____ Staffing of WBPHCOT

_____ Oversight and management of WBPHCOT

_____ Monitoring and evaluation of WBPHCOT activities

_____ Use of mHealth tools for WBPHCOT activities

_____ Impact of WBPHCOT on health services at the facility-level (*please specify*) ______

_____ Other (*please specify*) ____

_____ Don't know

A6. What are <u>the main factors that have facilitated</u> implementation of the expanded WBPHCOT activities? *(Select up to three items from the list below)*

_____ Prioritization by DOH

_____ Prioritization by PEPFAR/CDC/USAID

_____ Alignment with national policy

_____ Availability of national guidelines

_____ Availability of funding to support WBPHCOT activities

_____ Commitment of health facilities

_____ Support from patients/communities

_____ Availability of M&E tools for WBPHCOT

_____ Availability of training curriculum

_____ Other (*please specify*) ____

_____ Don't know

A7. What are <u>the main challenges</u> to the implementation of the expanded WBPHCOT activities? *(Select up to three items from the list below)*

_____ Lack of political prioritization by DOH

_____ Lack of prioritization by PEPFAR/CDC/USAID

_____ Lack of alignment with national policies

_____ Lack of availability of national guidelines

_____ Lack of funding to support WBPHCOT activities

_____ Lack of standardized compensation/payment scale for CHWs

_____ Lack of commitment of health facilities

_____ Lack of support from patients/communities

_____ Lack of availability of M&E tools for WBPHCOT

_____ Lack of training curriculum

_____ Other (*please specify*) ____

_____ Don't know

A8. What changes or modifications have been made to the expanded WBPHCOT activities since they were initially planned? *(Select all that apply)*

_____ No changes

_____ Changes to locations implementing, or served by, WBPHCOT

_____ Changes to the WBPHCOT staffing or management approach

_____ Changes to the scope of work of WBPHCOT

_____ Changes to the timeline for WBPHCOT expansion

_____ Other (*please specify*) ____

_____ Don't know

A9. What innovations—activities, ways of working, service delivery, other—have you observed during the implementation of the expanded WBPHCOT activities?

_____ *(Tick if no innovations)* □ Don't know (*tick*)

B. Results and Unintended Consequences

B1. On a scale of -5 to 5, where -5 is *large negative impact*, 0 is *no impact*, and 5 is a *large positive impact*, what impact would you say the expanded WBPHCOT activities have had so far in the following areas? *(Enter a value ranging from -5 to 5 for each of the three items below)*

_____ HIV testing coverage and timeliness □ Don't know (*tick*)

_____ Linkage to care/ART for individuals diagnosed with HIV □ Don't know (*tick*)

_____ Retention in care/ART □ Don't know (*tick*)

-5 -4 -3 -2 -1 0 1 2 3 4 5

Large negative impact | No impact | Large positive impact

B2. What <u>unintended consequences</u>, either positive or negative, have you observed during the implementation of the expanded WBPHCOT activities?

_____ *(Tick if no unintended consequences)* □ Don't know (*tick*)

B3. Is there anything <u>else</u> should we know about the expanded WBPHCOT activities that has not been covered in this interview?

C. Demographics

C1. How old were you on your last birthday?

C2. Gender

_____ Male

_____ Female

C3. What is your highest level of education?

_____ Some primary

_____ Completed primary

_____ Some secondary

_____ Completed secondary

_____ Some tertiary

_____ Completed tertiary

C4. For how long have you worked with WBPHCOT programs?

_____ < 1 year

_____ 1-5 years

_____ > 5 years and <10 years

_____ ≥10 years

_____ Don't know

D. Recommend Other Participants

Thank you for participating in this electronic questionnaire. We are looking for more participants from the national, district, and/or provincial level to complete the questionnaire. If you know someone who would be a good fit to participate in the evaluation, please share their name(s), contact information, and affiliation in the space below:

Thank you for your time.
