# Supplementary material for: "They are our eyes outside there in the community": Implementing enhanced training, management and monitoring of South Africa’s ward-based primary healthcare outreach teams
Source: PLoS One. 2022 Aug 26;17(8):e0266445. doi: 10.1371/journal.pone.0266445 (PMC9417004; doi:10.1371/journal.pone.0266445)
Supplement: S5 File — (PDF) [file pone.0266445.s005.pdf]

WBPHCOTKnowledge, Attitudes and Practices (KAP) Questionnaire

Please check this box to indicate that this study has been explained to you and that you have given your consent to participate ☐

Date: ________________________

District: ________________________

1. ABOUT YOU

Please select one best answer or all that apply (when indicated).

No.	Question	Response
1.	Are you a CHW or OTL?	1. CHW<br>2. OTL
2.	Do you identify as male or female?	1. Male<br>2. Female<br>3. Other<br>99. Decline to answer
3.	How old were you on your most recent birthday?	___________ years
4.	What is your highest level of education?	1. Some primary<br>2. Completed primary<br>3. Some secondary<br>4. Completed secondary<br>5. Some university<br>6. Completed university<br>99. Decline to answer
5.	What is your primary (home) language?	1. English<br>2. Zulu<br>3. Sotho<br>4. Sepedi<br>5. Tswana<br>6. Xhosa<br>7. Other<br>99. Decline to answer

No.	Question	Response
6.	[FOR CHWs ONLY]<br>When did you first start working as a CHW?	1. < 1 year ago<br>2. 1-5 years ago<br>3. > 5 years and <10 years ago<br>4. ≥10 years ago<br>88. Don't know<br>99. Decline to answer
7.	[FOR OTLS ONLY]<br>For how long have you been a nurse?	1. < 1 year<br>2. 1-5 years<br>3. > 5 years and <10 years<br>4. ≥ 10 years<br>88. Don't know<br>99. Decline to answer
8.	How long have you worked in your current district?	1. <1 year<br>2. 1-2 years<br>3. 3-5 years<br>4. > 5 years<br>88. Don't know<br>99. Decline to answer
9.	How long have you worked on your current ward–based outreach team?	1. <1 year<br>2. 1-2 years<br>3. 3-5 years<br>4. >5 years<br>88. Don't know<br>99. Decline to answer
10.	Have you received any training on WBPHCOTs?	1. Yes<br>2. No<br>99. Decline to answer<br><br>*IF NO, skip to question 18.*
11.	In what month and year was the most recent training?	[month/year]
12.	Who provided the recent WBPHCOT training?	1. Department of Health<br>2. I-TECH<br>3. Aurum<br>4. WRHI<br>5. Other NGO<br>6. Other, *please specify*: ________________<br>7. Don't know<br>99. Decline to answer
13.	Where did the training take place -- at your place of work, off-site, or both?	1. On-site<br>2. Off-site<br>3. Both<br>88. Don't know<br>99. Decline to answer

No.	Question	Response
14.	Which of the following health conditions and services were covered in the recent WBPHCOT training course?<br>*Read options to respondent and check all that apply*	1. Maternal health<br>2. Child health<br>3. HIV testing<br>4. HIV self-screening<br>5. HIV/AIDS treatment<br>6. TB screening<br>7. TB treatment<br>8. Hypertension diagnosis<br>9. Hypertension treatment<br>10. Diabetes diagnosis<br>11. Diabetes treatment<br>12. Mental health and/or substance abuse<br>13. Other *please specify*<br>88. Don't know<br>99. Declined to answer
15.	Which of the following activities and skills were covered in the recent WBPHCOT training course?<br>*Read options to respondent and check all that apply*	1. Health promotion<br>2. Household assessment<br>3. Referrals for HIV testing and/or other diagnostic services<br>4. Adherence support for clients on chronic medications (including but not limited to ART)<br>5. Defaulter tracking for clients who miss appointments<br>6. Counseling and communication skills<br>7. Coordinating community-based ART adherence groups<br>8. Documenting WBPHCOT activities<br>9. Interpersonal violence<br>10. Confidentiality and ethics<br>11. Health systems navigation<br>12. Other *please specify*<br>88. Don't know<br>99. Decline to answer

No.	Question	Response
16.	[FOR OTL ONLY] Did the recent WBPHCOT training course cover the following management skills?<br>*Read options to respondent and check all that apply*	1. Mentoring<br>2. Supportive supervision<br>3. In-service training of CHWs<br>4. Developing weekly and monthly reports<br>5. Using the data collected by the CHWs to assess team performance<br>6. Using the data collected by the CHWs to assess individual performance<br>88. Don't know<br>99. Declined to answer
17.	To what extent did the recent training prepare you for your current role as a CHW or OTL on a WBPCHOT?<br>*Read options to respondent and check one*	1. Not at all prepared<br>2. Somewhat prepared<br>3. Very well prepared<br>4. Completely prepared<br>88. Don't know<br>99. Declined to answer
18.	Did you take a post-test after your most recent training	1. Yes<br>2. No → SKIP to q20<br>1. Don't know → SKIP to q20<br>99. Declined to answer → SKIP to q20
19a.	What was your post-test score?	1. [score]<br>88. Don't know<br>99. Decline to answer
19.	Do you have a written job description, outlining your current roles and responsibilities?	1. Yes<br>2. No<br>88. Don't know<br>99. Decline to answer
20.	Have you received a formal evaluation of your work on the WBPHCOT in the past 12 months?	1. Yes<br>2. No<br>88. Don't know<br>99. Decline to answer
21.	[FOR OTLS ONLY]: Have you conducted any formal evaluation of CHWs in the past 12 months?	1. Yes<br>2. No<br>88. Don't know<br>99. Decline to answer

PLEASE PROCEED TO THE NEXT SECTION.

2. REFLECTIONS ON THE WBPHCOT

No	Question	Response
22.	In general, I am satisfied with my job on the WBPHCOT	1. Strongly agree<br>2. Somewhat agree<br>3. Somewhat disagree<br>4. Strongly disagree<br>88. Don't know<br>99. Decline to answer
23.	I am satisfied with my pay.	1. Strongly agree<br>2. Somewhat agree<br>3. Somewhat disagree<br>4. Strongly disagree<br>88. Don't know<br>99. Decline to answer
24.	I feel that my workload is manageable (not too burdensome)	1. Strongly agree<br>2. Somewhat agree<br>3. Somewhat disagree<br>4. Strongly disagree<br>88. Don't know<br>99. Decline to answer
25.	I consistently have the supervision I need to perform my duties.	1. Strongly agree<br>2. Somewhat agree<br>3. Somewhat disagree<br>4. Strongly disagree<br>88. Don't know<br>99. Decline to answer
26.	I consistently have the supplies I need to perform my duties.	1. Strongly agree<br>2. Somewhat agree<br>3. Somewhat disagree<br>4. Strongly disagree<br>88. Don't know<br>99. Decline to answer
27.	I consistently have access to the transportation resources needed to perform my duties	1. Strongly agree<br>2. Somewhat agree<br>3. Somewhat disagree<br>4. Strongly disagree<br>88. Don't know<br>99. Decline to answer
28.	If it were up to me, I would continue to work on this WBPHCOT for quite some time.	1. Strongly agree<br>2. Somewhat agree<br>3. Somewhat disagree<br>4. Strongly disagree<br>88. Don't know<br>99. Decline to answer
For the next questions, reflect on your perceptions of the WBPHCOT. Select the response that best reflects your opinion.		

No	Question	Response
29.	How would you rate the quality of community-based care we provide for people in this community? In general, would you say *poor*, *fair*, *good*, or *excellent*?	1. Poor<br>2. Fair<br>3. Good<br>4. Excellent<br>88. Don't know<br>99. Decline to answer
30.	In your opinion, how effective are the WBPHCOTs? Would you say *very effective*, *somewhat effective*, *somewhat ineffective*, or *very ineffective*?	1. Yes, very effective<br>2. Yes, somewhat effective<br>3. No, somewhat ineffective<br>4. No, very ineffective<br>88. Don't know<br>99. Decline to answer
31.	In your opinion, how well do the services offered by WBPHCOT fit with community needs and expectations? Would you say *poor*, *fair*, *good* or *excellent*?	1. Poor<br>2. Fair<br>3. Good<br>4. Excellent<br>88. Don't know<br>99. Decline to answer
32.	How confident are you in your ability to provide high-quality counseling about HIV testing and referrals? Would you say very *confident*, *somewhat confident*, *neutral* or *not confident*?	1. Very confident<br>2. Somewhat confident<br>3. Neutral<br>4. Not confident<br>88. Don't know<br>99. Decline to answer
33.	How confident are you in your ability to assist clients to link to HIV treatment if they test positive for HIV?	1. Very confident<br>2. Somewhat confident<br>3. Not very confident<br>4. Not at all confident<br>88. Don't know<br>99. Decline to answer
34.	How confident are you in your ability to provide high-quality medication adherence counseling for clients on chronic medications?	1. Very confident<br>2. Somewhat confident<br>3. Not very confident<br>4. Not at all confident<br>88. Don't know<br>99. Decline to answer
35.	How confident are you in your ability to track clients who have missed appointments and assist them to return to the health facility?	1. Very confident<br>2. Somewhat confident<br>3. Neutral<br>4. Not confident<br>88. Don't know<br>99. Decline to answer
36.	How confident are you in your ability to document your work on the WBPHCOT correctly, using the M&E tools?	1. Very confident<br>2. Somewhat confident<br>3. Neutral<br>4. Not confident<br>88. Don't know<br>99. Decline to answer

3. KNOWLEDGE ASSESSMENT

Lesson A: Ethics and Confidentiality

A1 SHORT ANSWER: List three examples of ethical behavior.

1.____

2.____

3.____

A2. Telling your mother that Tumi, the pregnant woman in one of the households you are working, just found out she is HIV-infected is a breach (breaking) of confidentiality.

a. True
b. False

A3. CHOOSE ALL THAT APPLY: Examples of sharing "Need-to-know" information includes:

a. Sharing medical information about a household member with their partner.
b. Sharing the name of a CHW's boyfriend with the OTL because he is HIV infected and not on ART.
c. Sharing household member information with the counselor who is facilitating an adolescent HIV support group.
d. Reporting abuse based on observations by the CHW of a grandmother beating a young child.

Lesson B: Communication

B1. CHOOSE ALL THAT APPLY: Effective verbal communication (talking) includes:

a. Giving definitions of clinical or technical words
b. Speaking clearly
c. Providing health education in the local language
d. Using local terms for body parts

B2. SHORT ANSWER: List two examples of effective **non-verbal** communication (using gestures or expressions with your face or body).

1.____

2.____

B3. Circle "open-ended" or "closed" for each of the following questions.

When did you last go to the clinic?	Open-ended	Closed
How did you get that bruise?	Open-ended	Closed
Do you have a headache?	Open-ended	Closed
How are you feeding your baby?	Open-ended	Closed
Are you giving only breast milk to your baby?	Open-ended	Closed

B4. CHOOSE ONE: When asking a question to a SHY household member:

a. Ask "Why won't you talk to me?"
b. Say "speak louder!"
c. Move to a quiet place and repeat the question.
d. Leave, because she won't answer your questions.

B5. CHOOSE ONE: When speaking with a CURIOUS household member:

a. Explain that you will be coming back regularly
b. Ask, "Why are you asking so many questions?"
c. Leave, because she is taking up too much time
d. Say, "Let me speak."

B6: CHOOSE ONE: When speaking with an UNFRIENDLY household member:

a. Ask, "Why are you so cruel?"
b. Leave and never come back.
c. Offer praise
d. Say, "You should be friendlier."

B7: CHOOSE ONE: When speaking with an AGGRESSIVE household member:

a. Shout
b. Ask, "What is wrong with you?"
c. Force your way inside the house.
d. Say, "I hear your complaints"

B8. SHORT ANSWER: List three communication "Don'ts".

1.____

2.____

3.____

Lesson C: Health Promotion

C1. CHOOSE ALL THAT APPLY: People are more likely to change if you:

a. Ask them to make small changes that will have a big effect.
b. Give them lots of information with pictures.
c. Give them options to consider and then choose what to do.
d. See others making positive changes.
e. Give lots of criticism.

C2. CHOOSE ALL THAT APPLY: Which of the following are examples of health promotion activities that the CHW should do?

a. Promote exclusive breastfeeding and good young child feeding practices
b. Promote accident prevention and safety in the home
c. Educate on basic hygiene and infection control including hand washing, safe food preparation
d. Provide blood tests and HIV test
e. Promote HIV prevention including HIV testing, condom use, male circumcision, STI treatment

C3. SHORT ANSWER: List three skills that a CHW needs in order to be a good health promoter.

1.____

2.____

3.____

C4. SHORT ANSWER: List three places where the CHW/OTL can use the Health Promotion Tool.

1.____

2.____

3.____

Lesson D: Screening

D1. CHWs can diagnose conditions like high blood pressure and diabetes.

a. True
b. False

D2. When using a screening tool, what is the final step?

a. Educate the client on the issue (area) just screened
b. Read "notes to the client"
c. Count the answers
d. Explain the purpose of the tool

D3. SHORT ANSWER: List three groups that should be targeted for screening.

1.____

2.____

3.____

Lesson E: Referrals

E1. CHOOSE ALL THAT APPLY: Which of the following are needed for a referral?

a. A completed Community Services Referral Form
b. Client must have been seen and assessed by a CHW or other health worker
c. The client must show a clear understanding of why there is a need for referral
d. In the case of a child, the parent/guardian or caregiver must give consent
e. In the case of an orphan, a next of kin must be provided

E2. If a household member needs to be referred to more than one place, you can tick multiple boxes on the referral form.

a. True
b. False

E3. CHOOSE ALL THAT APPLY: Barriers to referrals include:

a. Lack of time
b. Stigma
c. Distance
d. Confidentiality
e. Not understanding why the referral is needed

E4. SHORT ANSWER: List three ways to encourage people to use referrals.

____

____

____

Lesson F: Tracing

F1. CHOOSE ALL THAT APPLY: The CHW is responsible for working with the OTL to trace patients who have missed treatment and appointments for:

a. HIV
b. Anaemia
c. Child immunizations
d. TB

F2. CHOOSE ONE. Which of the following HIV+ community members should be prioritized <u>first</u> for tracing?

a. Recently tested but not yet initiated on ART
b. Early missed appointment (5-14 days)
c. Late missed appointment (15-30 days)
d. Lost to follow up (90 days or more)

F3: CHOOSE ONE: Our tracing motto is "the earlier the better," which means:

a. Trace everybody who has missed an appointment
b. Trace people lost a long time ago first.
c. Trace people lost most recently first.
d. Don't trace anybody.

F4. A child who has started an immunisation series but has not appeared within four weeks of the scheduled time to receive the next dose is considered lost to follow up.

a. True
b. False

F5. SHORT ANSWER: List three types of people who are at high risk of defaulting.

1.____

2.____

3.____

Lesson G: Psychosocial Support

G1. SHORT ANSWER: List three types of psychosocial support.

1.____

2.____

3.____

G2. CHOOSE ALL THAT APPLY: In order to show empathy, the OTL must be:

a. Open-minded
b. Sympathetic
c. Accepting of himself or herself
d. Committed

G3. CHOOSE ALL THAT APPLY: Which of the following are part of the counselling process?

a. Good listening
b. Summarising
c. Telling people what to do
d. Establishing a relationship
e. Monitoring behaviour

G4. Working with children and adolescents is the same as working with adults.

a. True
b. False

ONLY CHWs

Lesson H: Data Collection

H1. CHOOSE ALL THAT APPLY: Which of the following are needed in order to have data quality?

a. Integrity
b. Usability
c. Timeliness
d. Accuracy

e. Reliability

H2. SHORT ANSWER: List three possible causes of poor data.

____

____

____

ONLY OTLs

Lesson I: Supportive Supervision

I1. CHOOSE ALL THAT APPLY: In order to supervise effectively, I need:

a. Strong coordination and communication
b. To supervise my team on a daily basis
c. Bonus pay
d. To provide continuous training
e. Community involvement

I2. SHORT ANSWER: List three supervisory skills of an OTL.

1.____

2.____

3.____

I3. CHOOSE ALL THAT APPLY: Which of the following are needed for supervision visits?

a. Transport
b. Per diem
c. Adequate time
d. Copies of supervision checklists
e. HIV test kits

I4. CHOOSE ALL THAT APPLY: What should be documented in the record-book for supportive supervision visits?

a. Training given
b. Main observations
c. Follow-up plans
d. Household visit notes
e. Date of visit

Lesson J: Mentoring Skills

J1. SHORT ANSWER: Adults learn differently than children. Please list three principles of adult learning:

1.____

2.____

3.____

J2. CHOOSE ALL THAT APPLY: Which of the following are steps of the mentoring cycle?

a. Identify the team's training needs
b. Psychosocial support for CHW
c. Identity CHW training needs
d. Document activities
e. Onsite training

J3. A "teachable moment" happens only when the CHW asks a question.

a. True
b. False

J4. SHORT ANSWER: List three ways to mentor.

1.

____

2.

____

3.

____

Lesson K: Monitoring and Evaluation

MATCHING: Match the data process with the correct definition:

K1. AGGREGATING data	a. Sharing and making data available
K2. ANALYSIS of data	b. Checking the data are correct
K3. VERIFICATION of data	c. Putting individual pieces of data together
K4. DISSEMINATION of data	d. Determining what the data show us

K5. If the OTL collects, stores, and manages poor-quality data, it will have an on impact WBPHCOT planning.

a. True
b. False

K6. CHOOSE ALL THAT APPLY: Which of the following are needed in order to have data quality?

a. Integrity
b. Usability
c. Timeliness
d. Accuracy
e. Reliability

K7. SHORT ANSWER: List three possible causes of poor data.

1. ____

2. ____

3. ____

Lesson L: Enabling Ongoing Learning for CHWs

L1. SHORT ANSWER: List three reasons why time management is important.

1. ____

2. ____

3. ____

L2. The Work-Integrated Learning phase of training is on a fixed schedule, so the OTL must follow the program in order.

a. True
b. False

L3. CHOOSE ALL THAT APPLY: How can you identify knowledge and skill gaps of CHWs?

a. Clinic topic pre/post-tests

b. Input from team meetings
c. CHW Core Skills Assessment
d. I just know when they have gaps
e. CHW assessment of a household member with TB

L4. SHORT ANSWER: List three items that need to be presented in a case presentation.

1. ____

2.____
_

3. ____

Thank you very much for your time.
