# Supplementary material for: "They are our eyes outside there in the community": Implementing enhanced training, management and monitoring of South Africa’s ward-based primary healthcare outreach teams
Source: PLoS One. 2022 Aug 26;17(8):e0266445. doi: 10.1371/journal.pone.0266445 (PMC9417004; doi:10.1371/journal.pone.0266445)
Supplement: S6 File — (PDF) [file pone.0266445.s006.pdf]

WBPHCOT FIELD-BASED OBSERVATION DATA COLLECTION FORM

To the Observer:

Do not begin the interview unless all WBPHCOT team members being observed today have agreed to be part of the field-based observation

Did all WBPHCOT team members agree to participate?

If YES, please check box
If NO, please check box and STOP the field-based observation

Yes, all team members agreed	
No, at least one team member refused	

OBSERVER: PLEASE FILL OUT THIS SECTION (QUESTIONS 100-107) BEFORE BEGINNING TIME MOTION DOCUMENTATION.

100. Observer Code: ________________________________

101. Date: __ __ / __ __ / __ __

102. Name of District: ________________________________

103. Name of WBPHCOT Health Facility: ________________________________

104. Ward Number: ________________________

105. How many CHWs are on the team/being observed today? ___________________________

106. How many OTLs are on the team/being observed today? ____________________________

107. Is anyone else (other than yourself) accompanying the team today? ________

IF YES, *specify*: _________________________

Time Motion – Direct Observation

OBSERVER: RECORD EACH ACTIVITY OBSERVED AS A SEPARATE LINE

TIME-MOTION ACTIVITY CODES/TITLES

A. Logistics Planning
1. Planning outreach schedule for the day
2. Other planning and preparatory activities

B. Travel
1. Waiting for transportation or directions
2. In transit
3. Locating/identifying household (HH) or other meeting place

C. Household visits
1. Waiting for client to arrive and/or become available
2. Introduction of CHW, obtaining permission to enter HH, speaking to household members, etc.
3. Time spent in/with HH

D. Documentation
1. Completing paper-based forms
2. Entering data into electronic database or mHealth application
3. Other documentation activities

E. Meetings
1. WBPHCOT team meeting
2. Meeting with OTLs
3. Meeting with community-level stakeholders
4. Other meeting activities

F. Other community-based activities

G. Other facility-based activities

H. Lunch Break, Coffee Break, Rest

I. WAITING (other than specified in B1 and C1)
1. For equipment, medications, supplies
2. For people

J. OTHER, s*pecify*_______________

Add a new line of data each time an activity changes. Add as many rows as necessary. All time needs to be accounted for.

Observation Number	Activity Code (see above)	Activity Title	Team member Involved	# of Team Members Involved	Start Time	End Time	Notes (see examples)
Example:	*B*	*Travel*		*2*	*08:15*	*08:42*	*Visit #2 to house*
	C	*HH entry*		*1*	*08:42*	*08:45*	*Introductions, permission to enter*
	D	*Time spent in HH*		*1*	*08:45*	*09:15*	*CHW reports key activities were: BP check, adherence counselling (not directly observed) and that clients were all adults*
1							
2							
3							

Comments:

Reflection questions:

1. **What went well today?**

2. **Were there challenges with any of the following (*select all that apply*):**

O transportation
O equipment/supplies
O locating clients
O staff availability
O staff dynamics, *specify*_________________
O weather
O safety
O documentation/M&E forms
O contacting OTL or other supervisors
O other, *specify* _________________________
O no challenges

3. **Can you describe the specific challenges that you observed today?**

4. **What would help to address these challenges?**

Daily activity summary

How many household visits did the CHW(s) being observed make today? ________________________

Instructions: Repeat this section for each HH visit observed

For each HH visit, please obtain the following information from the CHWs:

- Is this a new or follow-up HH visit? [*select one*]
    - New = HH not visited by the WBPHCOT in the past 12 months
    - Follow-up = HH visited by the WBPHCOT at least once in the past 12 months
- What were the age categories of the clients who were visited/received services from the WBPHCOT? [*select all that apply*]
    - Infants
    - Children
    - Adolescents/young adults
    - Adults
- What services were provided by the CHWs being observed today? [*select all that apply*]
    - HIV related services
        - Counselling/education of clients with unknown HIV status
        - Referrals for HIV testing
        - Distribution of HIV self- testing kits
        - Performance of HIV rapid test
        - Adherence assessment for patients on ART (pill counts, structured self-report, symptom check, other)
        - Adherence support for patients on ART (education, motivational interviewing, other)
        - Medication delivery
        - Defaulter tracking for HIV patients who have missed an appointment (clinical, lab, drug pick-up)
        - Referrals for facility-based clinical, laboratory and/or psychosocial support
        - Referrals for community-based clinical, laboratory and/or psychosocial support
        - Counselling/education (safer sex, condom use, family planning, other)

    - Food and nutrition support
    - Condom distribution
    - Other HIV-related services, *specify*________________________
- TB-related services
    - Symptom screening (e.g., structured questions)
    - Referrals for TB testing
    - Counselling/education (cough etiquette for patients with chronic cough)
    - Adherence *assessment* for TB patients on treatment and/or people on TB preventive treatment (TPT)
    - Adherence *support* for TB patients on treatment and/or people on TB preventive treatment (TPT)
    - Defaulter tracking for TB patients who have missed an appointment (clinical, lab, drug pick-up)
    - Other TB-related services, *specify*________________________
- Reproductive health services
- Maternal child health services
- Non-communicable disease (NCD) services (hypertension, diabetes)
- Other, *specify*________________________
