# Supplementary material for: "They are our eyes outside there in the community": Implementing enhanced training, management and monitoring of South Africa’s ward-based primary healthcare outreach teams
Source: PLoS One. 2022 Aug 26;17(8):e0266445. doi: 10.1371/journal.pone.0266445 (PMC9417004; doi:10.1371/journal.pone.0266445)
Supplement: S7 File — (PDF) [file pone.0266445.s007.pdf]

Appendix I: Facility Assessment Tool

WBPHCOT Process Evaluation

Structured Facility Assessment Tool

ID for Staff completing Assessment		
Assessment Date		
Assessment Time	Start:	Finish:

Facility Identification and Details

Facility Name	
Facility Level	1. PHC<br>2. CHC
Subdistrict	1. East<br>2. North<br>3. South
Neighborhood population density	1. High Density<br>2. Medium Density<br>3. Low Density
Facility Location	1. Rural<br>2. Peri-urban<br>3. Urban
Facility Size: Number of inpatient beds	
Facility Size: Number of OPD visits/month	
Are HIV treatment services supported by an implementing partner(s)?	1. No<br>2. Yes, *specify implementing partners*
Are any non-HIV health services supported by other implementing partners?	1. No<br>2. Yes, *specify implementing partners*:
Are any research studies or projects taking place at the facility?	1. No<br>2. Yes, *specify type of research and who is implementing the study(ies)*:
Is any other additional support being provided to the health facility (e.g. campaigns, QI projects, etc.)?	1. No<br>2. Yes, *specify type of additional support, implementer, and funder*:

Health Facility HIV Program Data

Number of patients currently on ART	1. All males_________<br>1a. Age < 15 years______<br>1b. Age 15+ years______<br><br>2. All females______

	2a. Age < 15 years______<br>2b. Age 15+ years______
Number of patients newly initiating ART in previous month	1. All males_________<br>1a. Age < 15 years______<br>1b. Age 15+ years______<br><br>2. All females______<br>2a. Age < 15 years______<br>2b. Age 15+ years______
Number of patients tested for HIV in previous month	1. All males_________<br>1a. Age < 15 years______<br>1b. Age 15+ years______<br><br>2. All females______<br>2a. Age < 15 years______<br>2b. Age 15+ years______
Number of patients tested <u>positive</u> for HIV in previous month	1. All males_________<br>1a. Age < 15 years______<br>1b. Age 15+ years______<br><br>2. All females______<br>2a. Age < 15 years______<br>2b. Age 15+ years______
Number of ART (early, late) missed appointments in previous month	1. Early<br>2. Late
Number of ART (early, late) missed appointment clients returned to care in previous month	1. Early<br>2. Late
Number of patients who had viral load testing in previous 6 months	
Number of viral load tests that were < 400 copies/mL in previous 6 months	
Number of male condoms distributed in previous month	
Number of female condoms distributed in previous month	
Number of days in which HIV test kits were stocked out in previous month	

WBPHCOT Program Data

Number of home visits made by the team in past month	
Number of defaulter tracking visits made by the team in past month	

Appendix I: Facility Assessment Tool

M&E System for WBPHCOT

Are the new/revised M&E Tools in place?	1. Household Record: Yes ____ No____<br>2. Referral Form: Yes____ No _____<br>3. CHW Diary: Yes ____ No ____<br>4. Follow up Appointment Card: Yes ____ No____<br>5. CHW Daily Activity Tracker/Sheet: Yes____ No____<br>6. CHW Weekly Activity Sheet: Yes ____ No ____<br>7. CHW Monthly Activity Sheet: Yes ____ No ____<br>8. PHC OTL Monthly Activity Summary Form: Yes ____ No ____<br>9. Supervisor Assessment Form: Yes ____ No ____<br>10. HTS register: Yes____ No_____
Is the site using m-health tools to document WBPHCOT performance indicators?	1. No, paper-based only<br>2. Yes, CommCare paper (field) + electronic (facility)<br>3. Yes, CommCare, all electronic<br>4. Yes, HISP paper (field) + electronic (facility)<br>5. Yes, HISP, all electronic<br>6. Yes, other, *specify*______________
Is supportive supervision provided by DOH for collection, aggregation, and/or analysis of **WBPHCOT** data?	1. No<br>2. Yes, *specify for which activities, who from DOH provides it, and how frequently*______________
Is supportive supervision provided by any other organizations for collection, aggregation, and/or analysis of **WBPHCOT** data?	1. No<br>2. Yes, *specify for which activities, what organization provides it, and how frequently*__________________
Who aggregates the **WBPHCOT** data weekly and monthly at the facility?	**PROBE for** position of this individual
Has this facility been provided access to DHIS2 dashboards summarizing recent **WBPHCOT** data?	1. No<br>2. Yes, via on-site computer<br>3. Yes, via paper printouts
Does the OTL routinely use the M&E data to provide feedback to the CHWs as a group?	1. No<br>2. Yes, *describe frequency of feedback, feedback process, give an example*____________
Does the OTL routinely use the M&E data to provide feedback to individual CHWs?	1. No<br>2. Yes, *describe frequency of feedback, feedback process, give an example*_____________

Are there written **WBPHCOT** M&E standard operating procedures (SOPs) available at the facility?	1. No<br>2. Yes, by report only<br>3. Yes, observed by evaluation staff
Are there M&E related job aides available at the facility?	1. No<br>2. Yes, by report only<br>3. Yes, observed by evaluation staff

WBPHCOT Characteristics (Staffing & Training)

When was the new I-TECH WBPHCOT curriculum adopted by the facility?	[month/year]
How many **WBPHCOT** staff have left work at the facility since [DATE FROM ABOVE] (e.g., reposted to a different facility, resigned/fired, on leave)	1. [Number]<br>2. *Specify reasons for departure* if known___________________
How many **WBPHCOT** staff have been hired since [DATE FROM ABOVE]?	1. [Number]
Number of OTL actively working on **WBPHCOT** at time of assessment	
Percent of OTL trained on new (I-TECH) curriculum since [DATE FROM ABOVE]	
Number of CHWs actively working on **WBPHCOT** at time of assessment	
Percent of CHW trained on new (I-TECH) curriculum since [DATE FROM ABOVE]	
Number of data capturers exclusively assigned to **WBPHCOT**	

WBPHCOT Resources (Management)

Where do **WBPHCOT** meet for supportive supervision with OTL?	1. Designated office space. d*escribe*_____<br>2. Other space, *specify*____________
What equipment and supplies are available for the OTL and CHWs?	
What equipment and supplies are needed but are not available/missing for the OTL and CHWs?	
Is mobile phone airtime provided to **WBPHCOT** staff?	1. No<br>2. Yes, *specify how much is provided and how often* ______________________
What kind of transportation is provided to **WBPHCOT** teams to enable them to make household and community visits?	1. None<br>2. Bicycles<br>3. Motorbikes<br>4. Car/taxi<br>5. Other, *specify* _________________
Are job aides provided to **WBPHCOT** to guide their community-based activities?	1. No<br>2. Yes, *specify*_____________________

Data Quality Assessments

Review 4 recent (within the past month) entries from CHW Activity Sheets for 3 different CHW working on the **WBPHCOT** team (for a total of 12 entries).<br><br>For each of these entries, retrieve and review the corresponding Household Record - assessing whether the CHW Activity Sheet accurately reflects the activities and data in the Household Record for that visit.
Of the 12 entries reviewed (4 entries for 3 different CHW)<br>1. Number of entries accurately reflecting the activities and data in the Household Record:<br>2. Number of entries with inaccuracies:<br>3. Number of Household Records that could not be found:<br>Comments regarding inaccuracies:
Recount totals for 1 CHW Weekly Summary Sheet within the past month for 3 different CHW working on the **WBPHCOT** team.<br><br>Use CHW Daily Activity Sheets for that week for each CHW to calculate totals for the CHW Weekly Summary Sheet.
Of the records reviewed:<br>1. Number of CHW Weekly Summary Sheets accurately reflecting Daily Activity Sheets:<br>2. Number of CHW Weekly Summary Sheets with inaccuracies:<br>3. Number of CHW Daily Activity Sheets that could not be found:<br>Comments regarding inaccuracies:
Recount totals for 1 CHW Monthly Summary Sheet within the past 3 months for 3 different CHW working on the **WBPHCOT** team.<br><br>Use CHW Weekly Summary Sheets for that month for each CHW to calculate totals for the CHW Monthly Summary Sheet.
Of the records reviewed:<br>1. Number of CHW Monthly Summary Sheets accurately reflecting Weekly Summary Sheet:<br>2. Number of CHW Monthly Summary Sheets with inaccuracies:<br>3. Number of CHW Weekly Summary Sheets that could not be found:<br>Comments regarding inaccuracies:
Recount totals for the most recent PHC OTL Monthly Activity Summary Form. Use the CHW Monthly Summary Sheets for facility CHW under **WBPHCOT** for this month to calculate the totals.
1. Number of fields in PHC OTL Monthly Activity Summary Form that accurately reflect the totals across facility CHW Monthly Summary Sheets for the month<br>2. Number of fields in PHC OTL Monthly Activity Summary Form with inaccuracies<br>3. Number of CHW Monthly Summary Sheets that could not be found

Comments regarding inaccuracies:

Compare the most recent CHW data in DHIS2 for this facility with the corresponding data in the PHC OTL Monthly Activity Summary Form for this same month.

1. Number of fields in DHIS2 that accurately reflect the totals in the PHC OTL Monthly Activity Summary Form
2. Number of fields in DHIS2 with inaccuracies
3. Number of PHC OTL Monthly Activity Summary Form that could not be found

Comments regarding inaccuracies:

Identify 3 supervisor visits to observe CHW outreach over the past 2 months and retrieve Supervisor Assessment Forms for each visit.

1. For how many of these 3 visits are Supervisor Assessment Forms available? ________
2. Were available Supervisor Assessment Forms (as applicable) completed as expected? No/Yes
   a. If no, explain how forms were not used as expected:
