# Supplementary material for: "They are our eyes outside there in the community": Implementing enhanced training, management and monitoring of South Africa’s ward-based primary healthcare outreach teams
Source: PLoS One. 2022 Aug 26;17(8):e0266445. doi: 10.1371/journal.pone.0266445 (PMC9417004; doi:10.1371/journal.pone.0266445)
Supplement: S1 Table — (PDF) [file pone.0266445.s008.pdf]

Table A. Data collection strategy by sample and assessment domains.		
**Data Collection Strategy**	**Sample**	**Illustrative Domains**
Key informant interviews with policy and program stakeholders (in person, 1 hour)	Policy and program stakeholders with experience in the expanded OT activities (e.g., district-level DoH staff, implementing partners)	-OT program context and timeline<br>-Implementation successes and challenges<br>-Unintended consequences<br>-Best practices & key lessons learned
Online surveys with policy and program stakeholders	Policy and program stakeholders with experience in the expanded OT activities (e.g., district-level DoH staff, implementing partners)	-Implementation successes and challenges<br>-Unintended consequences<br>-Changes made to the initial implementation plan<br>- Innovations observed during implementation
In-depth interviews with facility-level staff (in person, 1 hour)	Facility Managers, OTLs, lay counselors and data clerks from the 20 health facilities	-Experiences with expanded OT program<br>-Perceptions of roles and responsibilities<br>-Perceptions of impact of OTs in the community<br>-Implementation successes and challenges<br>-Best practices & key lessons learned
Focus group discussions (in person, 2 hours)	CHWs from the 20 health facilities (6-10 participants per group)	-Views on OT program<br>-Perceptions of roles and responsibilities<br>-Impact of OTs in the community<br>-Implementation successes and challenges<br>-Remaining barriers and next steps
KAP surveys (self-administered, in-person, 45 minutes)	CHWs and OTLs from the 20 health facilities	-Satisfaction with OT training and preparedness for CHW/OTL role post-training<br>-Satisfaction with services provided and work environment<br>-Knowledge test
Field-based observation/time-motion study (2 hours)	Observations of OTs at the 20 health facilities using a structured observation checklist	-Amount of time spent planning for the day and meeting with team members and stakeholders

		-Amount of time walking from facility to a patient's home<br>-Amount of time to complete a full home visit<br>-Key activities undertaken during home visits<br>-Challenges and successes
Site assessments	20 health facilities	-HIV program data<br>-OT program data (staffing, training, management, M&E systems)<br>-Data quality assessments
Secondary data review of HIV services using DATIM (PEPFAR) data	The 20 purposively selected health facilities in the City of Tshwane and Bojanala Districts	HIV linkage, treatment, retention
Secondary data review of I-TECH pre- and post-test training scores	All CHWs and OTLs trained at 20 participating sites	Knowledge test<br>-Ethics & confidentiality<br>-Communication<br>-Health promotion<br>-Screening, referrals & tracing<br>-Psychosocial support<br>-Data collection (CHWs only)<br>-Supportive supervision (OTLs only)<br>-Mentoring skills (OTLs only)<br>-M&E (OTLs only)<br>-Enabling ongoing learning for CHWs (OTLs only)
